# Newt A1 cell-derived extracellular vesicles promote mammalian nerve growth

**DOI:** 10.1038/s41598-023-38671-z

**Published:** 2023-07-22

**Authors:** Ryan C. Middleton, Ke Liao, Weixin Liu, Geoff de Couto, Nahuel Garcia, Travis Antes, Yizhou Wang, Di Wu, Xinling Li, Warren G. Tourtellotte, Eduardo Marbán

**Affiliations:** 1 Smidt Heart Institute, Cedars-Sinai Medical Center, 8700 Beverly Blvd #2900A, Los Angeles, CA 90048 USA; 2 Gecorp, Av Juan Manuel de Rosas 899, San Miguel del Monte, Buenos Aires, Argentina; 3 Applied Genomics, Computation and Translational Core, Cedars-Sinai Medical Center, 8700 Beverly Blvd #2900A, Los Angeles, CA 90048 USA; 4 Department of Pathology, Cedars-Sinai Medical Center, 8700 Beverly Blvd #2900A, Los Angeles, CA 90048 USA

**Keywords:** Genetic vectors, Extracellular signalling molecules, Cell signalling, Cellular neuroscience, Adult neurogenesis

## Abstract

Newts have the extraordinary ability to fully regenerate lost or damaged cardiac, neural and retinal tissues, and even amputated limbs. In contrast, mammals lack these broad regenerative capabilities. While the molecular basis of newts’ regenerative ability is the subject of active study, the underlying paracrine signaling factors involved remain largely uncharacterized. Extracellular vesicles (EVs) play an important role in cell-to-cell communication via EV cargo-mediated regulation of gene expression patterns within the recipient cells. Here, we report that newt myogenic precursor (A1) cells secrete EVs (A1EVs) that contain messenger RNAs associated with early embryonic development, neuronal differentiation, and cell survival. Exposure of rat primary superior cervical ganglion (SCG) neurons to A1EVs increased neurite outgrowth, facilitated by increases in mitochondrial respiration. Canonical pathway analysis pinpointed activation of NGF/ERK5 signaling in SCG neurons exposed to A1EV, which was validated experimentally. Thus, newt EVs drive neurite growth and complexity in mammalian primary neurons.

## Introduction

During embryonic development, mammals retain the ability to regenerate neural and cardiac tissues and even entire digits, but this ability is lost shortly after birth^1,2^. Some animals, such as newts and other salamanders, can regenerate organs and tissues, including the spinal cord, into adulthood^3–5^. An understanding of the signaling pathways involved in newt regeneration may assist in developing therapies for human nerve regeneration following injury. For newts, the first step in replacing damaged tissue involves covering and closing the margin of injury by wound epithelium^6–8^. Next, the epithelium is infiltrated by neurites that direct the formation of the blastema, or stem cell cap, which directs cellular proliferation, and then differentiation into mature tissues^9–11^. Paracrine signaling factors play an important role in driving nerve-dependent blastema formation and nerve growth into the wound epithelium^12,13^. Extracellular vesicles (EVs) are among the candidate paracrine factors under investigation. EVs are nanometer scale-sized vesicles that are secreted from all cells, and contain many different varieties of RNA, protein and lipids^14^. These paracrine signaling factors can modulate the morphology and behavior of recipient cells, and, depending on EV contents, may promote cell proliferation, tissue vascularization, nerve regeneration and immune modulation^15–17^. We have previously shown that myogenic precursor cells (A1 cells), isolated from skeletal muscle of Eastern Newts (*Notophthalmus viridescens*), secrete EVs that protect mammalian cardiomyocytes from oxidative stress through the activation of the PI3K/AKT pathway^18^. Analysis of the RNA cargo of A1-derived EVs (A1EVs) yielded a surprising number of messenger RNAs (mRNAs) involved in neural differentiation and development^18^. These findings motivated our present investigation into how cultured mammalian primary neurons respond to A1EVs.

## Results

### In silico analysis of newt A1EV cargo identifies mRNA involved in neural growth and development

**Figure 1:**
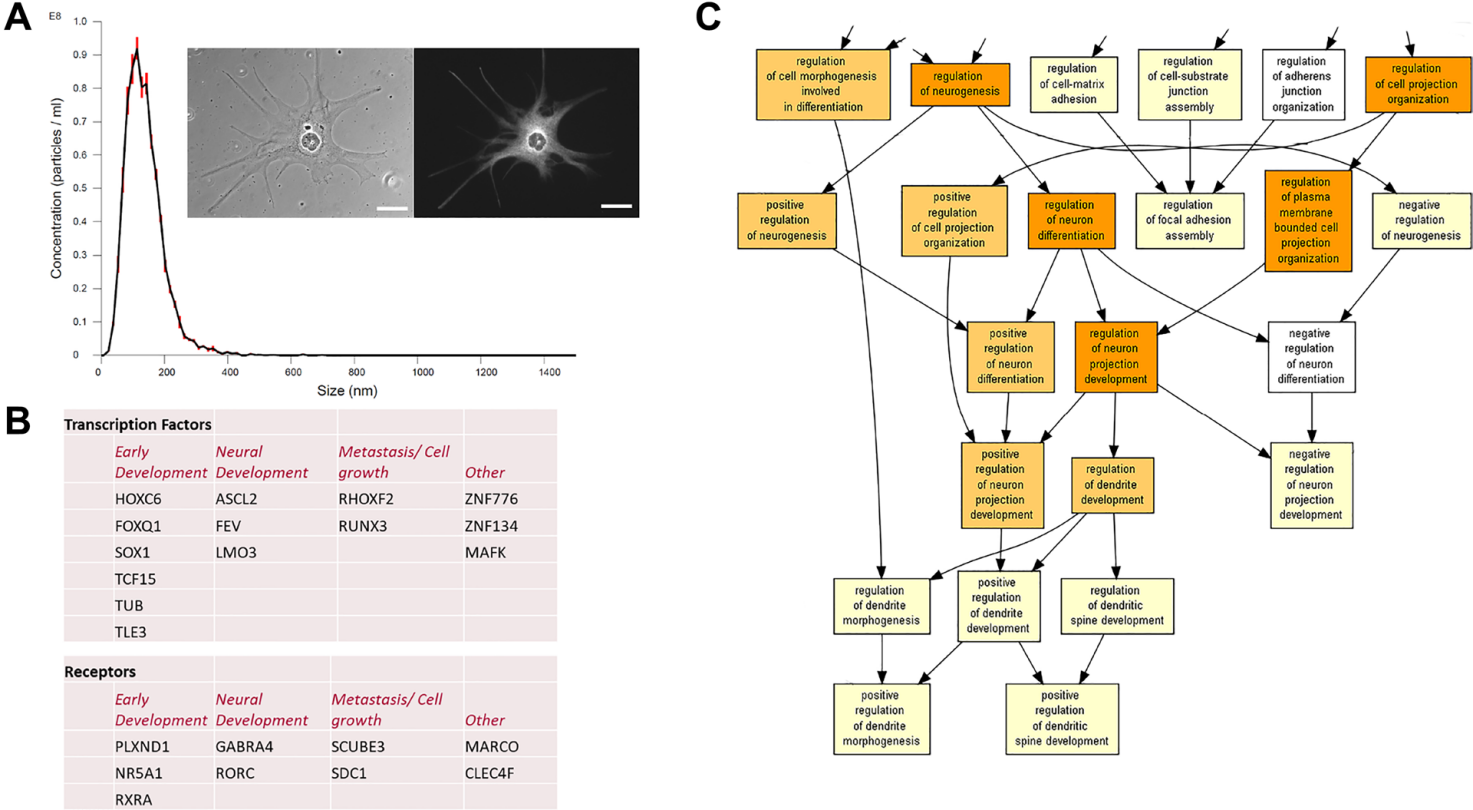
In Silico analysis of newt A1EV cargo identifies genes involved in neural growth and development. (**A**) Dynamic light scattering of A1EVs isolated from A1 cells (inset) show the size distribution of EVs. A1 cells are shown as imaged by phase-contrast microscopy (left inset) and confocal microscopy following staining against the keratin blastema marker, 22/18. Scale bar = 10 µm. (**B**) Table displaying transcription factor and cell receptor mRNAs associated with embryonic and neural development, discovered by in silico analysis of RNA cargo within A1EVs. (**C**) Gene Ontology analysis of all mRNAs identified within the A1EV cargo, ranked by abundance. A portion of the Cellular Function diagram associated with neuron differentiation and neurite growth is shown.

EV cargos are key mediators of functional regulation in recipient cells. In our previous study, EV RNA cargo, isolated from newt A1 cells (Fig. 1a), was profiled by RNA-Seq and mapped using sequence similarity to human genome databases^18^. As shown in Fig. 1b, we had identified many unique mRNAs in newt A1EVs not found within the EV cargo of two mammalian cell lines, a cardiac-derived stem cell and normal human dermal fibroblasts. Closer scrutiny of mRNA unique to A1EVs revealed many of these mRNAs are involved in early embryonic development and neuronal differentiation. Gene Ontology Analysis (GOA) identified mRNA involved in several biological processes related to neuron function, including neuron projection growth and development; p-value = 2.93E−23 and 1.54E−19, respectively (Fig. 1c, Supplemental Fig. 1 and Supplemental Table 1). Additionally, much of the mRNA cargo generated gene expression products that are commonly found within the cellular components of the neuron projections and synapses; p-values = 1.67E−21 and 4.75E−23, respectively (Supp. Fig. 2 and Supp. Table 2).

### A1EVs drive neurite-like extensions in cell lines that model nerve growth

To determine if A1EVs can affect neuron differentiation or drive neurite growth, 1 × 10^8^ A1EVs were added to cultured PC-12 cells, a rat pheochromocytoma line of the adrenal medulla that expresses the nerve growth factor (NGF) receptor and respond to NGF by generating plasma membrane projections that share similarities to neurites. After 48 h of incubation with either A1EVs, or A1 cell culture media in which EV generation had been suppressed (20 µM GW4869), or media-only control, the PC-12 cells displayed many more neurite-producing cells with A1EV incubation than with the media control or with A1 media lacking EVs. (Supp. Fig. 3).

### A1EVs promote neurite growth and branching in mammalian sympathetic neurons, in a dose-dependent manner

**Figure 2:**
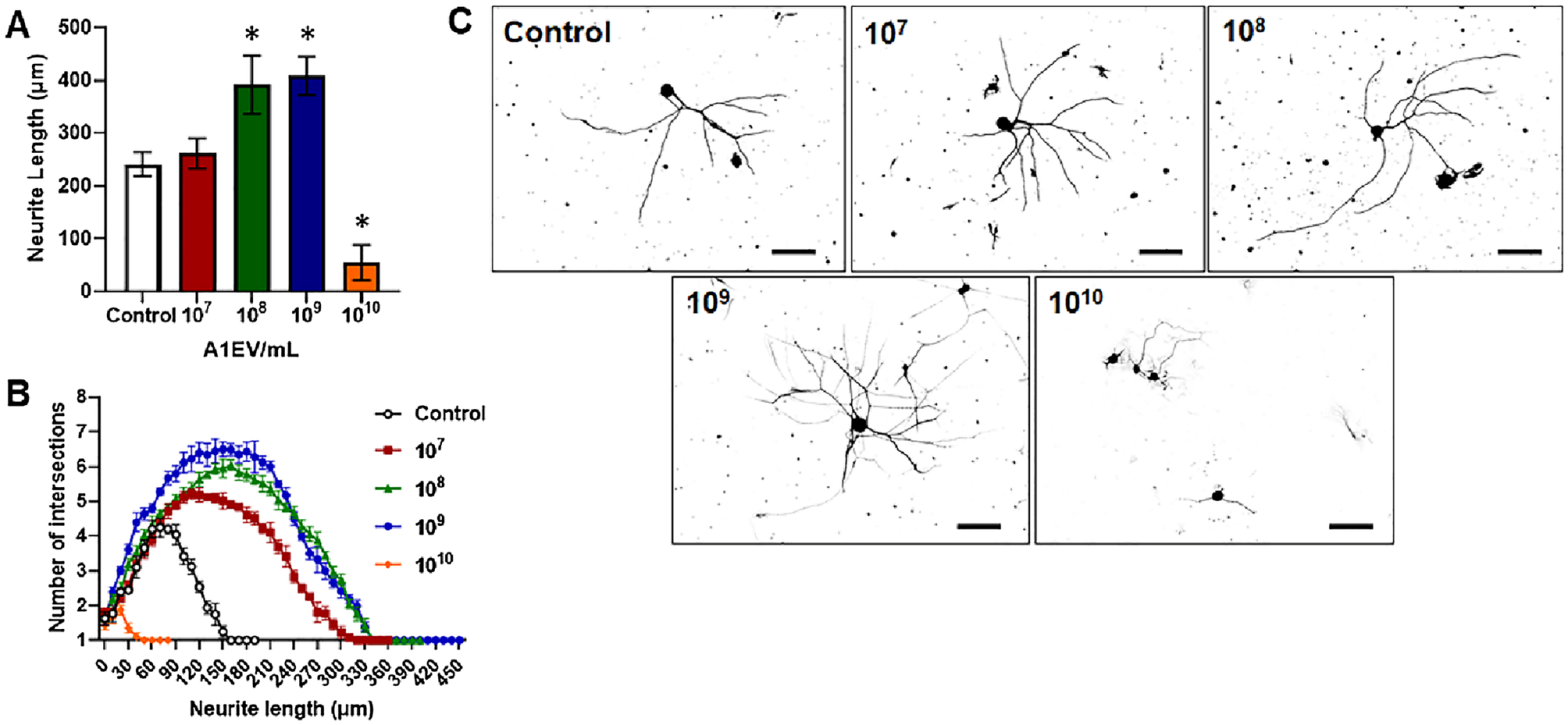
A1EVs promote neurite growth and branching in mammalian sympathetic neurons, in a dose-dependent manner. Graphical representations of the (**A**) average neurite length and (**B**) neurite complexity, 12 h after exposure to increasing concentrations of A1EVs. (**C**) Neurite silhouettes, as imaged by confocal microscopy, demonstrate neurite growth at different concentrations of A1EVs (indicated at the top left of each image, in particles per milliliter). Scale bar = 100 µm. * Indicates statistical significance from Control cells, p < 0.01.

As PC-12 cells are not true neurons^19,20^, the potential nerve growth properties of A1EVs were tested on sympathetic neurons derived from rat superior cervical ganglions (SCG). Sympathetic neurons were chosen as a study model amongst other nerve types as sympathetic neuron growth and neurite development require minimal growth factors that may mask or confound the effects of A1EVs. Neonatal rat SCG neurons were plated sparsely to avoid inter-cell contact and multiple cultures were exposed to increasing concentrations of A1EVs and incubated for 12 h. As described in the methods section, the A1EVs were thoroughly washed in PBS prior to addition into the neuron culture to remove any secreted factors from the A1 cells that are not part of the A1EV cargo, such as Nerve Growth Factor (NGF), that could confound the effects of the A1EVs. Alpha acetylated tubulin staining revealed increases in neurite length after 12 h, which corresponded to increases in A1EV concentration. 1 × 10^8^ and 1 × 10^9^ A1EVs/mL yielded the maximum neurite length (Fig. 2a,c). Interestingly, at 1 × 10^10^ EVs/mL, very little neurite growth occurred, far less than in the media-only control group. Sholl analysis^21^, a measure of neurite complexity, of A1EV-treated neurons indicated that the average neurite growth and the number of neurite intersections also increases with increasing concentration of A1EV, up until the 1 × 10^10^ EVs/mL concentration, which showed little neurite growth and complexity (Fig. 2b,c). Similar effects on neurite growth were also demonstrated on sensory DRG neurons (Supp. Fig. 4). Since both 1 × 10^8^ and 1 × 10^9^ EVs/mL showed strong effects on the induction of neurite growth, 1 × 10^8^ EVs/mL was chosen for all the ensuing experiments.

### The rate of neurite growth increases following A1EV exposure, but growth cone number and length are similar to untreated control

**Figure 3:**
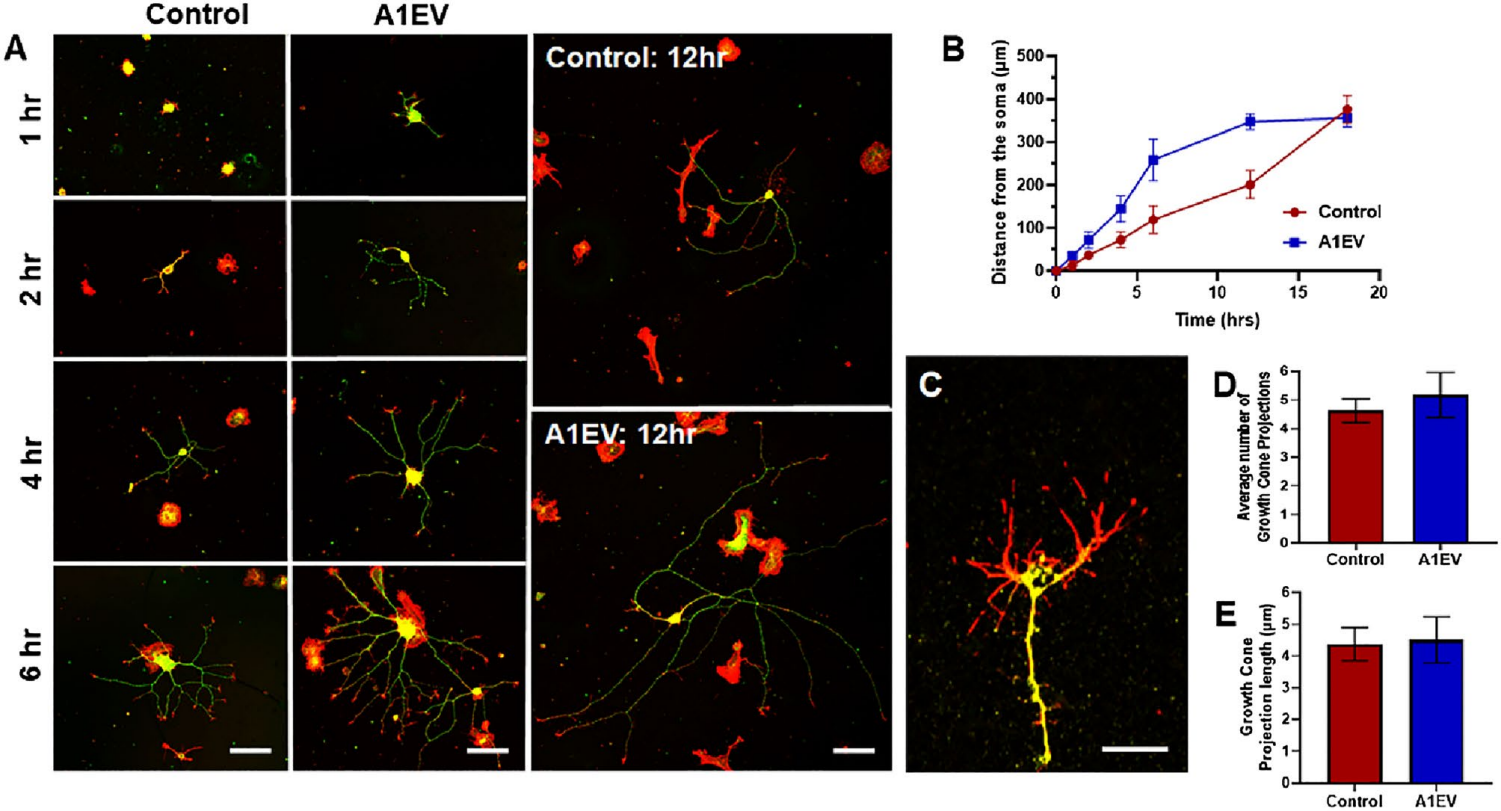
The rate of neurite growth increases following A1EV exposure, but number and length of growth cones remain similar to control. (**A**) Confocal images of sympathetic neurons taken at 2-, 4-, 6- and 12-h post A1EV exposure. Alpha acetylated tubulin (green) and filamentous actin (red) are shown. Scale bar = 100 µm. (**B**) Graphical representation of average neurite length following A1EV or media control exposure. (**C**) High magnification (63 ×) confocal image of neurite growth cone, as indicated by positive filamentous actin staining (red) and negative alpha acetylated tubulin staining (green). Scale bar = 5 µm. Graphical representations of average growth cone number (**D**) and average growth cone projection length (**E**) are shown.

Next, to determine the optimal time for A1EV-mediated induction of neurite growth, SCG sympathetic neurons were administered 1 × 10^8^ A1EVs/mL or media alone, and neurite growth was assessed 1-, 2-, 4-, 6-, and 12-h post-exposure. Enhanced neurite growth occurred within one hour of A1EV treatment, indicating that A1EVs may activate endogenous growth pathways via receptor signaling, as opposed to translation of delivered mRNA cargo (Fig. 3a,b). Although there appears to be a sudden decrease in neurite growth rate in A1EV-treated neurons at 12 h, this was likely due to the neurites coming into contact with one another. Growth cone length and number of projections were assessed by measuring the length and number of neurite tips that stained positive for filamentous actin (phalloidin), but negative for alpha acetylated tubulin. No significant difference was observed between A1EV-exposed and unexposed neuron cultures (Fig. 3c–e).

### A1EV suppression diminishes mammalian neurite growth

**Figure 4:**
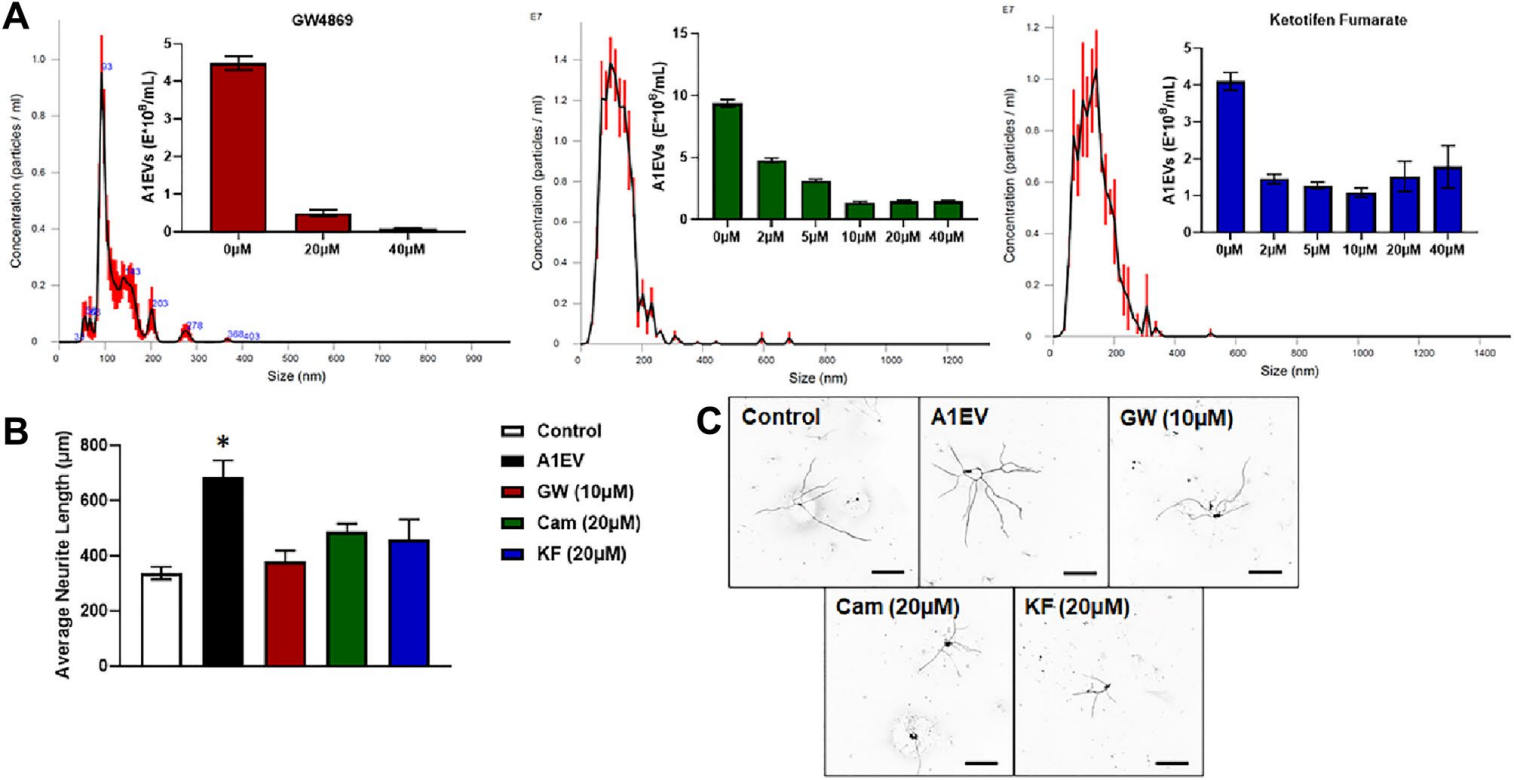
A1EV suppression diminishes mammalian neurite growth. (**A**) Dynamic light scattering graphs indicate the number and size distribution of A1EVs in the culture media of A1 cells treated with increasing concentrations of EV synthesis inhibitors: GW4869, cambinol, and ketotifen fumarate. (**B**) Average neurite length of sympathetic neurons following treatment with 1 × 10^8^ A1EVs/mL or equivalent volumes of media alone or A1 culture media that had been exposed to effective concentrations of EV synthesis inhibitors. (**C**) Confocal images of neurite silhouettes at 12 h post-treatment. Scale bar = 100 µm. * Indicates statistical significance from Control cells, p < 0.05.

To confirm that A1EVs were responsible for the enhanced neurite growth, and not some other secreted, non-vesicle bound factors, we added EV generation inhibitors (e.g., GW4869, an inhibitor of ceramide synthesis which suppresses A1EV secretion^18^) to A1 cells in culture. To establish effective inhibitor concentrations, A1 cells were exposed to increasing concentrations of GW4869 and two other EV inhibitors (cambinol, and ketotifen fumarate) concurrently with plating of the A1 cells (Fig. 4a). The lowest effective concentrations for A1EV suppression for GW4869, cambinol or ketotifen fumarate were 20 µM, 10 µM and 10 µM, respectively. For the following experiment, primary SCG neurons were plated in complete media and then one hour after treatment, the neurons were exposed to MEM only (control), 1 × 10^8^ A1EVs/mL in MEM, or equivalent volumes of the MEM collected from inhibitor-exposed A1 cultures. As described in the methods section, the enriched A1EV MEM media was thoroughly washed in PBS during the ultrafiltration step to remove any remaining EV synthesis inhibitors that could affect neuron outgrowths. Neurons were cultured for 12 h, then fixed and analyzed by immunohistochemistry and confocal microscopy. While A1EV-exposed neurons demonstrated enhanced neurite growth, those neurons that received few or no A1EVs did not grow significantly faster than the MEM-only control neurons (Fig. 4b).

### Fluorescently labelled A1EVs generation and neuron uptake

**Figure 5:**
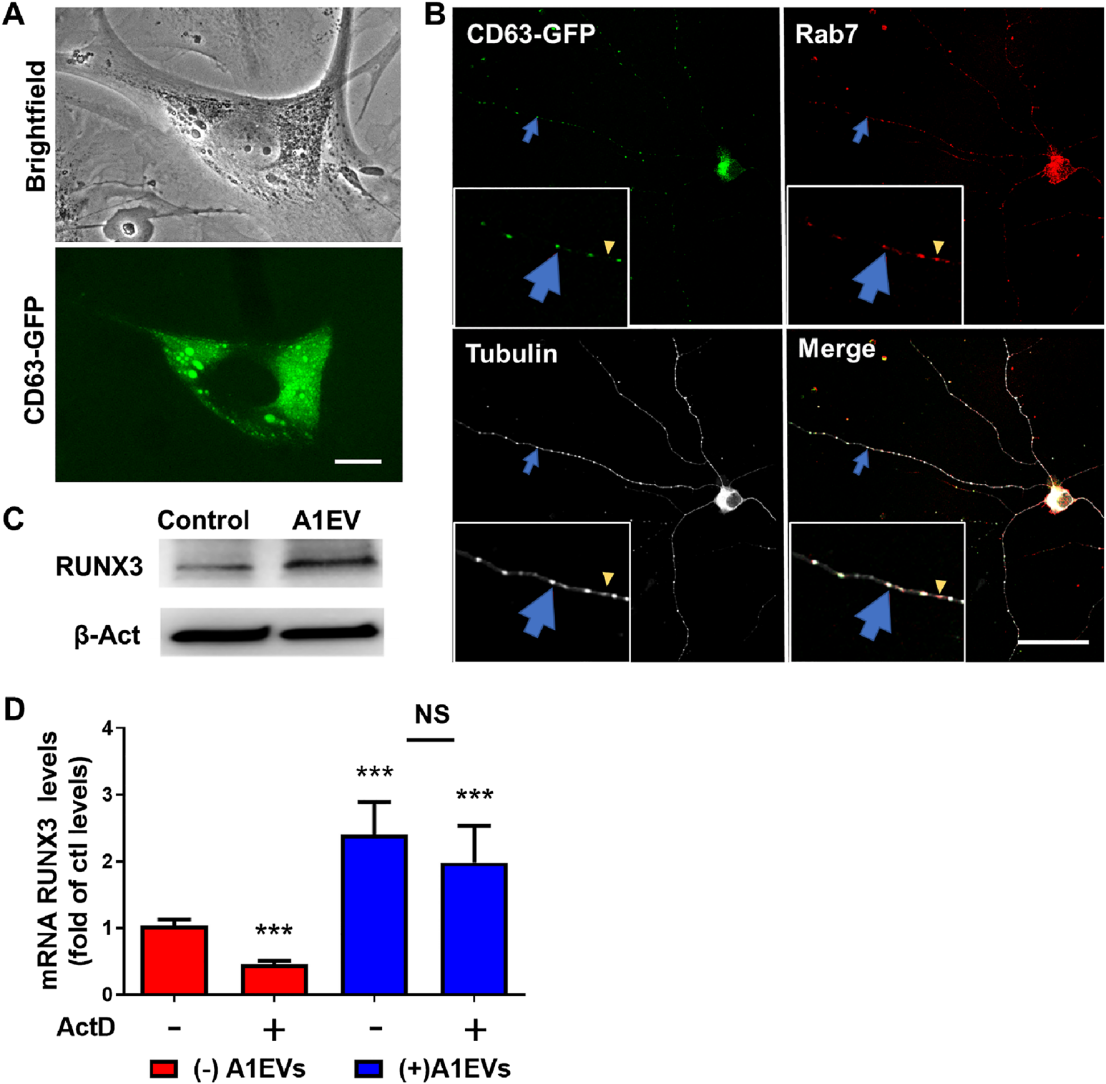
Fluorescently labelled A1EV generation and neuron uptake and RUNX3 expression. (**A**) Brightfield and confocal images of A1 cells following transfection of the CD63-GFP fusion protein plasmid. The green cytoplasmic signal shows the expression of the green fusion protein embedded in the EV membrane, with the larger, brighter green masses likely indicating the localization of EVs within multivesicular bodies as the EVs form. Scale Bar = 10 µm. (**B**) Confocal images of alpha acetylated tubulin-stained neurons (white) show the colocalization of the GFP-labelled A1EVs (green) with the endocytosis marker, Rab7 (red). The large blue arrows found within the images, or 10 × magnified insets, show the colocalization of A1EVs with Rab7 within the neurite, while the small yellow arrowheads show Rab7 alone. Scale Bar = 50 µm. (**C**) Western blots showing RUNX3 expression in A1EV or media control-treated neurons at 12 h post treatment. (**D**) Graphical representation of RUNX3 expression in SCG neurons, following exposure to A1EVs, with and without actinomycin D (ActD) pretreatment, as assessed by qPCR from isolated SCG neuron RNA. *** Indicate statistical significance from experimental condition without A1EVs treatment or ActD pretreatment.

To track A1EVs in solution and assess uptake by cultured neurons, we generated an A1EV cell line that expressed a CD63-GFP fusion protein. CD63 is a tetraspanin protein commonly used as a cell surface marker to identify and isolate EVs for study. Phase-contrast and fluorescent microscopy images of transfected A1 cells, show diffuse, cytosolic GFP expression as well as concentrated pockets of CD63-GFP within the Golgi (Fig. 5a). Two hours after exposure of cultured sympathetic neurons to the fluorescent A1EVs (5 × 10^8^ A1EV/mL), co-localization of GFP-labelled A1EVs with Rab7, a late endosomal marker that co-localizes with internalized EVs^22^ was observed along the length of the neurites (Fig. 5b).

A substantial body of evidence demonstrated that EV-resident mRNAs can be translated into functional proteins in recipient cells^23^. Several mRNAs involved in early embryonic development and neuronal differentiation, such as RUNX3, are enriched in A1EVs (Fig. 1b). RUNX3 is a critical factor in regulating early outgrowth of neurons^24,25^. While RUNX3 mRNA was enriched within the A1EVs, no RUNX3 protein was found within the EVs^18^. The next logical step then was to examine whether RUNX3 mRNAs residing in A1EVs can be translated and contribute to protein expression in recipient neurons. Analysis of protein expression, via Western Blot, in SCG neurons found significantly higher levels of RUNX3 following A1-EV exposure compared to media-only controls (Fig. 5c). To assess whether the increase in mRNA of the RUNX3 gene observed in A1EVs-treated SCG neurons was dependent on the delivery of mRNAs by A1EVs, or on transcription of endogenous RUNX3 mRNA within the recipient cells, we pretreated SCG neurons with the transcriptional inhibitor, actinomycin D (ActD) (Fig. 5d). The expression of RUNX3 was significantly upregulated in the presence of A1EVs, and that upregulation remained unaffected by ActD, indicating that mRNA transfer from A1EVs directly contributed to the increased expression of RUNX3. Moreover, ActD was effective at the dosage used, as it successfully reduced the expression of RUNX3 in actinomycin D-treated cells in the absence of A1EVs. Collectively, these data provide robust evidence supporting the direct transfer of RUNX3 mRNA from A1EVs to SCG neurons.

### Enhanced neurite growth is supported by increased mitochondria and increased cellular respiration in A1EV-exposed neurons

**Figure 6:**
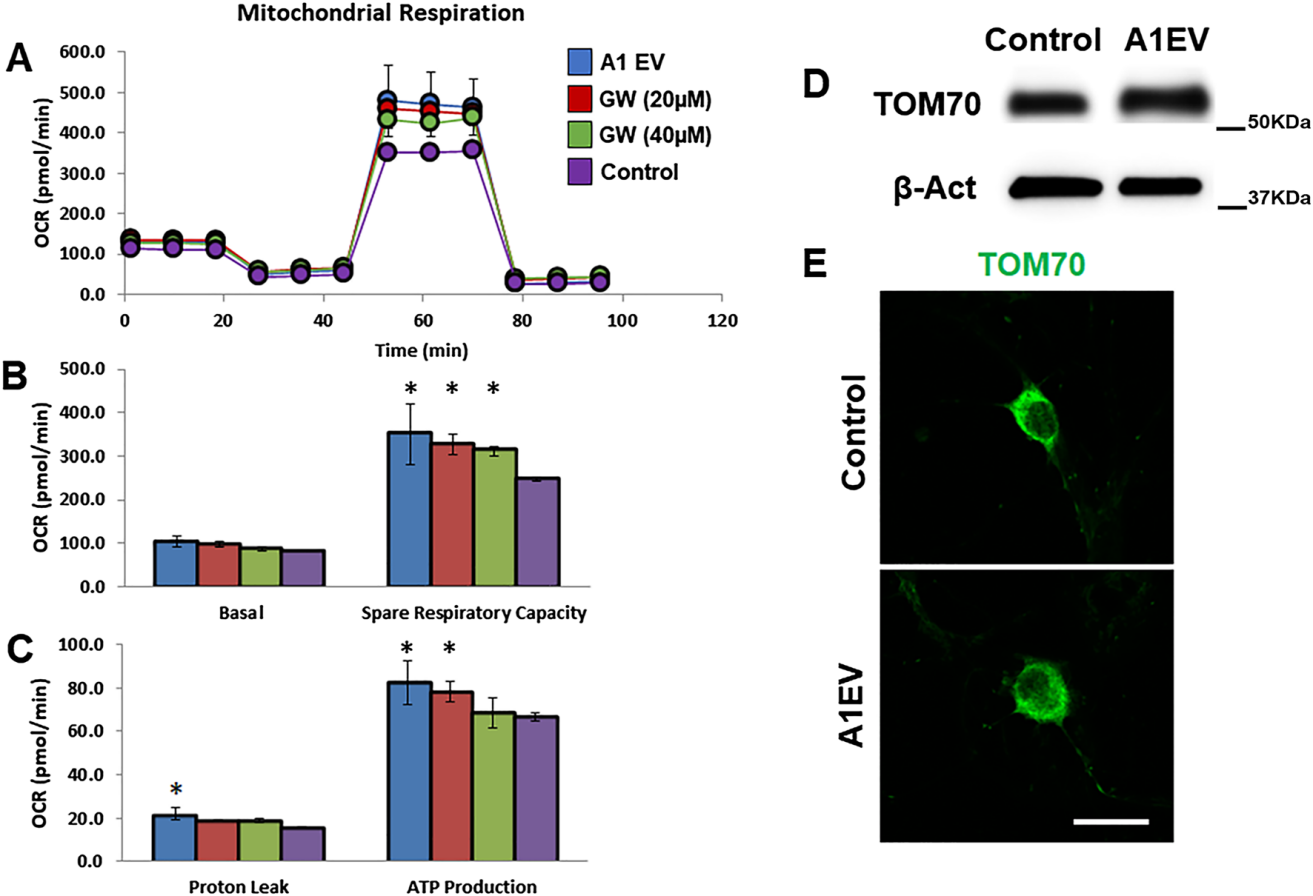
Enhanced neurite growth is supported by increased mitochondria and increased cellular respiration in A1EV-treated neurons. (**A**) Oxygen consumption rates (OCRs) of neurons treated with A1EVs, media alone, or EV-suppressed control media (GW4869: 20 µM and 40 µM). Focused analysis of basal respiration rates and spare respiratory capacity (**B**) and proton leak and ATP production are also shown (**C**). (**D**) Western blot analysis of the expression of the mitochondrial marker TOM70 in neurons following 12 h of A1EV or media only-treated neurons, to indicate mitochondria abundance. (**E**) Confocal images demonstrating TOM70 localization within A1EV or media only-treated neurons. Scale bar = 25 µm. * Indicates statistical significance from Control cells, p < 0.05.

The metabolic costs of facilitating higher neurite growth rates must be met with increased cellular respiration. Therefore, we measured oxygen consumption rates (OCR) of cultured SCG neurons exposed to A1EVs (1 × 10^8^/mL), equivalent volumes of media only (control), or A1 culture media lacking A1EVs (20 µM and 40 µM GW4869) (Fig. 6). Spare respiratory capacity and ATP production at baseline were significantly increased in A1EV-exposed neurons compared to media-only treated cells (Fig. 6a). Additionally, as shown in Fig. 6b,c, a concentration-dependent response was observed between increasing numbers of A1EVs (decreasing GW4869 concentration) and basal respiration, spare respiratory capacity, proton leak, and ATP production. Probing for the mitochondrial marker, TOM70, by Western blot in A1EV-exposed neurons (1 × 10^8^/mL) revealed increased mitochondrial staining compared to media-only control (Fig. 6b), but no changes in mitochondrial localization within A1EV-treated neurons (Fig. 6c).

### Canonical pathway analysis and validation of the IPA-predicted activation of NGF/ERK5 signaling pathway in SCG neurons treated with A1EV

**Figure 7:**
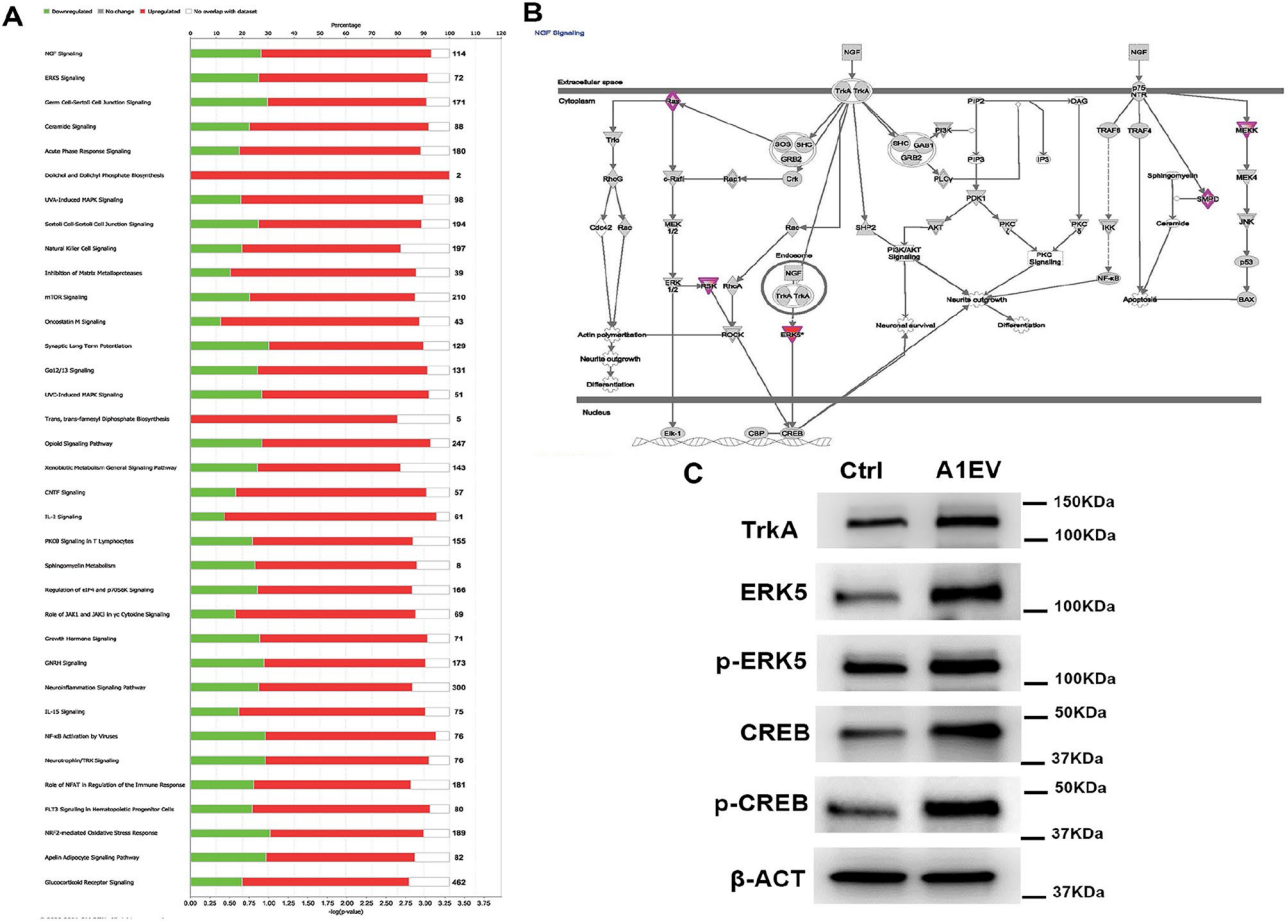
Canonical pathway analysis and the validation of the IPA-predicted activation of NGF/ERK5 signaling pathway in SCG neurons treated with A1EVs. (**A**) Ingenuity pathway analysis (IPA) of mRNA isolated from the A1EVs show the top 35 signaling pathways modulated by A1EV exposure, along with the activation score (Z-score) and the number of genes involved in each pathway. (**B**) Diagram of the NGF signaling pathway is shown that demonstrates the relationship between NGF/ERK5 signaling, PI3K/AKT signaling and neurite growth. (**C**) Western blot analysis of the protein expression and phosphorylation status of the major NGF/ERK5 signaling proteins in neurons treated with A1EV or a media-only control.

Having demonstrated that A1EVs could increase neurite growth, we sought to investigate the underlying molecular mechanisms. When comparing RNA from neurons exposed to 1 × 10^8^ A1EVs/mL or media only, sequencing data and Ingenuity Pathway Analysis (IPA) identified 36 significantly-altered canonical pathways (Fig. 7a). The top 2 regulated pathways were NGF signaling (− log(p-value) = 3.87) and ERK5 signaling (− log(p-value) = 2.43). For NGF signaling, 66% (75/114) of the pathway genes were upregulated, and 27% (31/114) downregulated. ERK5 signaling was also activated, with 65% (47/72) of the pathway genes upregulated and 26% (31/114) downregulated. Proteomic analysis of isolated A1EVs did not identify NGF protein within the EV cargo and RNA sequencing found negligible levels of NGF mRNA transcript (53 reads in 10 million)^18^ (Supplemental tables).

To validate IPA-predicted activation of NGF/ERK5 signaling pathway in SCG neurons exposed to A1EVs, we examined key proteins within the NGF signaling pathway (TrkA, ERK5 and CREB) (Fig. 7b) by Western Blot. The expression levels of TrkA, ERK5 and CREB were significantly upregulated in the A1EV-treated neurons compared to media-only controls (Fig. 7c). Overexpression of ERK5 has been demonstrated to strengthen ERK5 signaling^26,27^. The next logical step was to examine whether A1EV-mediated upregulation of ERK5 promoted downstream signaling activation by checking phosphorylation of ERK5 and CREB. Intriguingly, A1EV exposure also resulted in increased phosphorylation of ERK5 and CREB. These findings demonstrate that A1EV uptake activates NGF/ERK5 pathway in SCG neurons.

### Apoptosis is reduced following A1EV exposure in NGF-deprived SCG neurons

In addition to governing neurite outgrowth, NGF signaling pathway activation also regulates neuronal survival through downstream activation of PI3K/AKT growth and survival pathways^28^. Depravation of NGF in SCG neurons activates the mitochondrial pathway of apoptosis through BCL-2-associated X protein (BAX) and the release of cytochrome c. We had previously described the pro-survival effects of A1EVs in mammalian cardiomyocytes undergoing oxidative stress through the activation of PI3K/AKT signaling, but we did not determine if the benefits were due to cell receptor activation or through the incorporation of A1EV cargo components into the cells that stimulate signaling pathways downstream of the receptor.

**Figure 8:**
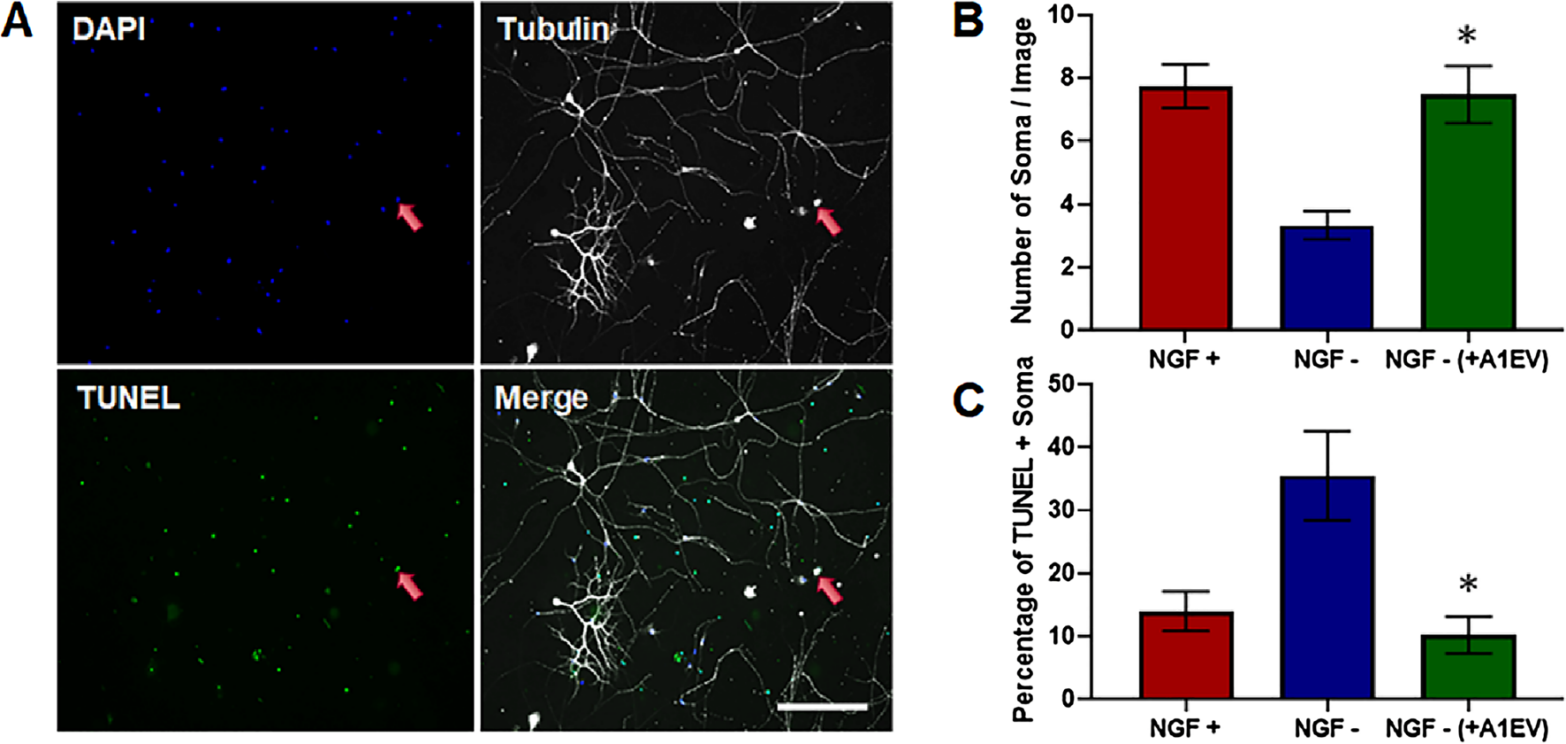
Apoptosis is reduced following A1EV exposure in NGF-deprived SCG neurons. (**A**) Examples of confocal images of cultured SCG neurons deprived of NGF for 12 h. The red arrow identifies the location of TUNEL-staining (green) within the nucleus (blue) of a single dying soma (white). Graphical representations of the number of soma (**B**) and number of TUNEL-positive soma (**C**) in the presence or absence of NGF and A1EVs. Scale bar = 150 µm. * Indicates statistical significance from NGF—neurons, p < 0.05.

To examine whether A1EV-mediated NGF signaling could promote survival, SCG neurons were cultured for 48 h in complete neural media + 10 ng/mL of NGF, then rinsed and switched to NGF-free media. Concurrently with the media change, neurons were exposed to 1 × 10^8^ A1EVs/mL or culture media only and cultured for an additional 12 h without NGF.. Neuron death was assessed by TUNEL staining, and by the number of neuron cell bodies (soma) present, an indicator of neuron apoptosis (Fig. 8a–c). The number of TUNEL-positive neurons was significantly decreased in A1EV-exposed cultures as compared with control. Additionally, tubulin staining revealed higher numbers of somas in NGF-depleted neuron cultures following A1EV exposure, compared to media-only control. Thus, A1EVs reduced primary neuron apoptosis induced by NGF withdrawal.

### Blocking the TRKA receptor does not inhibit A1EV-mediated neuron survival in NGF-depleted cultures

**Figure 9:**
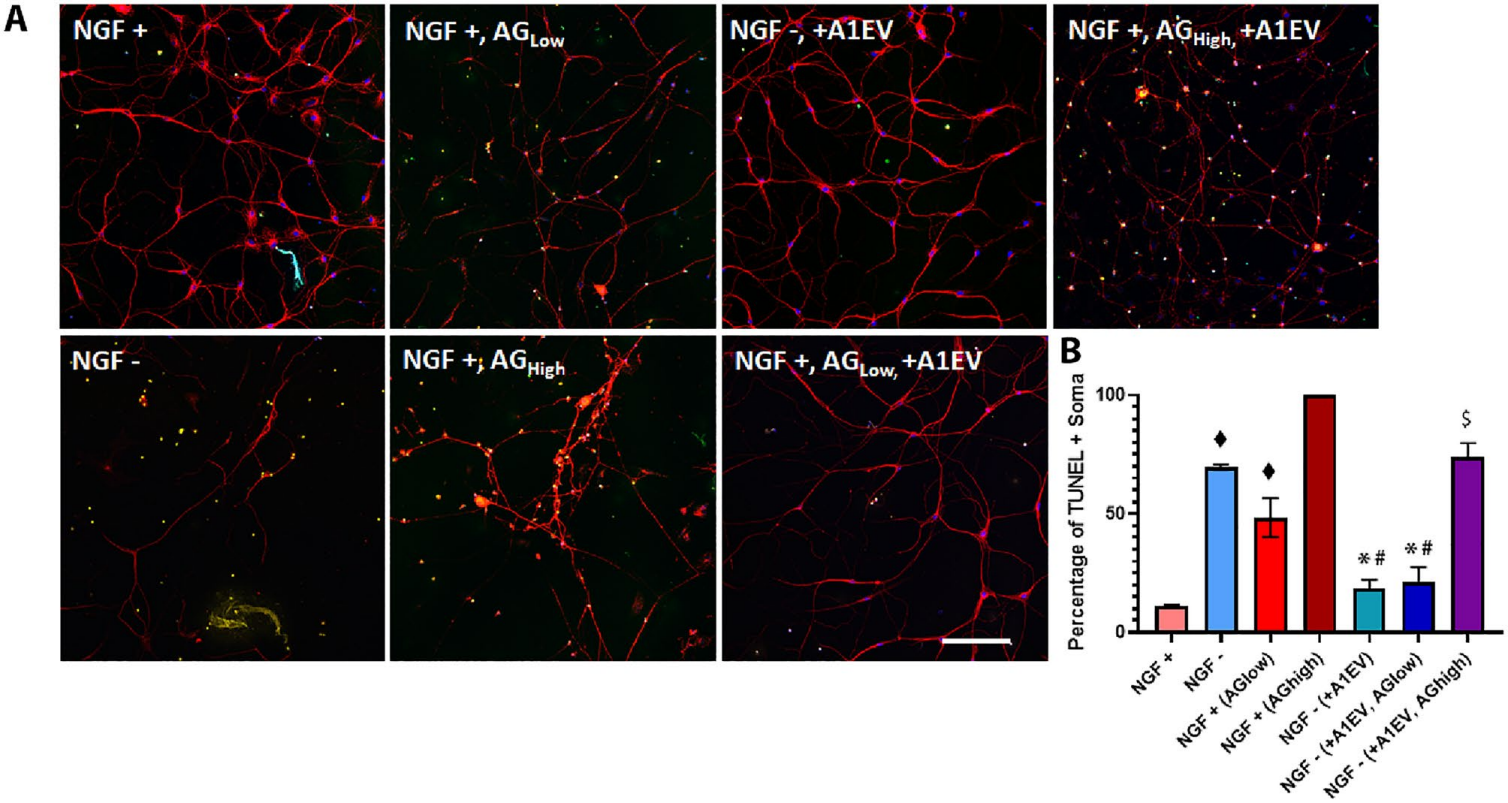
Blocking the TRKA receptor does not inhibit A1-EV-mediated neuron survival in NGF-depleted cultures. (**A**) Example confocal images of cultured SCG neurons (red) that identify TUNEL staining (green) localized to the nuclei (blue) under different treatment conditions. Scale bar = 150 µm. (**B**) Graphical representation of the number of TUNEL-positive neurons following 12 h of culture in the presence or absence of NGF, A1EVs and different concentrations of the TRKA-specific inhibitor, AG879: AGlow = 100 nM and AGhigh = 5 µM. ◆ Indicates statistical significance from (NGF+) neurons, p < 0.05. * Indicates a statistical significance from NGF—neurons, p < 0.01. # Indicates a statistical significance from NGF—(+AGlow) neurons, p < 0.05. $ Indicates a statistical significance from NGF+ (+AGhigh), p < 0.05.

Although Fig. 5b shows that internalized A1EVs co-localize with the Rab7 endocytic marker, it was unclear if A1EVs could also drive NGF/ERK5 signaling through activation of the Tropomyosin Receptor Kinase A (TRKA) receptor, the primary receptor of the NGF signaling pathway. To probe this question, primary SCG neurons were exposed to the TrkA receptor-specific inhibitor AG879 at two concentrations: 100 nM, which partially inhibits NGF-promoted survival and 5 µM, which fully blocks NGF-promoted survival^29,30^ (Fig. 9a,b). SCG neurons cultured in complete neural media + NGF maintained a basal apoptosis rate of approximately 11%. 12 h in NGF-depleted media resulted in increased TUNEL staining (71%), as expected. The addition of 100 nM AG879 (AGlow) and 5 µM AG879 (AGhigh) in the presence of NGF resulted in an increase of TUNEL-positive neurons (49% and 100%, respectively). The presence of A1EVs in the media without NGF rescued the neurons from apoptosis (19% vs. 71%), while neurons in NGF-depleted media, plus A1EVs and AG879 (AGlow), exhibited fewer TUNEL+ neurons than controls (21% vs. 71%). High levels of AG879 (AGhigh) + A1EVs in NGF-depleted cultures resulted in a partial rescue, with a 29% decrease in TUNEL-positive staining compared to SCG cultures treated with 5 µM AG879, that lack NGF. This indicates that, while A1EVs can protect neurons from NGF-depleted culture conditions, the mechanism can occur independent of the TrkA receptor. Thus, A1EV-mediated survival of neurons is accomplished through the NGF signaling pathway but independently of the TRKA receptor.

## Discussion

Newts are masters of regeneration. Even as mature adults, this salamander species has developed the ability to completely repair and regenerate tissues from organs including the spinal cord, brain, retina, and even entire limbs^3,6,7,11^. A new understanding of how secreted paracrine factors regulate the regenerative response in newts could provide further insight into regeneration in mammals and may translate to future therapies in humans. The molecular basis for the complete regeneration of injured tissues has been well studied in some amphibian species^7^. However, the paracrine signaling factors that orchestrate newt tissue repair are largely uncharacterized, and the conservation of these factors in other animals is not well defined. EVs serve as important players mediating cell-to-cell communication via EV cargo-mediated regulation of gene expression patterns in recipient cells. Recently, EVs have become an attractive research avenue due to their regenerative potential in many human tissues and organs, including nerve^16^, brain^31^, heart^18,32^, liver^33^, kidney^34^, cartilage^35^, muscle^36^, and dental pulp^37^. Previously, we had demonstrated that salamander cells produce EVs, which can be taken up by mammalian cardiomyocytes, leading to PI3K/AKT signaling pathway activation and ultimately conferring cytoprotective effects on mammalian cardiomyocytes^18^. Here, we demonstrate that A1EVs are taken up by mammalian neurons, resulting in enhanced neurite growth, in a dose-dependent manner, as well as neuron survival in the absence of nerve growth factor. Interestingly, there is a concentration limit to which A1EVs promote neurite growth. At 1 × 10^10^ A1EVs/mL, neurite growth is suppressed, perhaps indicating some level of toxicity of the A1EVs. Such bimodal effects, with paradoxical or negative effects at higher concentrations, have been described previously with various types of EVs^38–40^. However, additional investigation is required to determine the mechanism of neurite growth suppression with high A1EV concentrations. Additionally, we found increased mitochondria number and increased cellular respiration following A1EV exposure, supporting the increased neurite growth observed.

Assessment of OCR, with the use of electron transport chain inhibitors and uncoupling reagents to isolate specific contributors to the total OCR, reveals significant increases in spare respiratory capacity and ATP production in A1EV-exposed neurons. This modulation of oxygen consumption rates directly correlates to the concentration of A1EVs delivered to the neurons, as determined using EV synthesis inhibitors in A1 cells. Spare respiratory capacity, a measure of the difference between basal and maximal ATP production under oxidative phosphorylation^41^, is regulated through activation of the NGF signaling pathway and induces mitochondrial remodeling and increased mitochondrial biogenesis in many cell types^42,43^. Our studies reveal significantly higher levels of both ATP production and mitochondria in A1EV-treated sympathetic neurons.

A substantial body of evidence demonstrated that EV-resident mRNAs can be translated into functional proteins in recipient cells^23^. Analysis of the RNA cargo of the A1EVs yielded a surprising number of mRNAs involved in neural differentiation and embryonic development. This is fascinating as the A1 cell line is derived from newt skeletal muscle, as evidenced by the most enriched mRNA being MyoD, yet much of the mRNA content generates gene products that are most commonly associated with the cellular components of neurons and synapses. Among the enriched A1EVs mRNA, the gene RUX3 is considered a critical determining factor in early outgrowth of neurons^24,25^. Ken-ichi Inoue et al. found proprioceptive DRG axons disappear in Runx3^−/−^ mice, which, in turn, leads to severe ataxia^44^. Additionally, ex vivo studies by this group found that NT-3-responsive neurites from Runx3^−/−^ DRGs were significantly reduced in length. Therefore, we tested whether A1EVs could induce neurite outgrowth and found that SCG neurons exposed to A1EVs resulted in the upregulation of RUX3 expression as well as enhanced neurite outgrowth.

To further investigate the underlying molecular mechanisms by which A1EVs mediate neurite outgrowth, we isolated mRNA from neurons following treatment with A1EVs or a media control. RNA sequencing and IPA pinpointed the NGF/ERK5 signaling pathway as the most significantly changed by A1EV exposure. Several studies have demonstrated that activation of TrkA, upon NGF binding, leads to downstream ERK5 signaling activation, and this action prevents neuronal apoptosis^45–47^. Additionally, ERK5 expression is required for survival and development of neurons^48,49^. Intriguingly, our findings show that A1EVs could induce upregulation of RUX3 in neurons, and that TrkC and TrkA receptors were both positively regulated by RUNX3 in neurons^50^. IPA also indicated that A1EVs resulted in the upregulation of RUNX3, which increased expression of TrkA and contributed to the activation of NGF/ERK5 signaling pathway. This, in turn, led to neurite outgrowth and neuronal survival. Protein expression analysis confirmed that A1EVs induced increased TrkA, ERK5 and CREB expression, as well as phosphorylation of ERK5 and CREB. As noted in the results, NGF itself, was not found within the A1EV cargo. Sympathetic neurons require the presence of NGF to prevent neuron apoptosis^51,52^. In the absence of NGF, A1EV-treated neuron cultures showed decreased numbers of TUNEL-positive neurons as compared with media-only controls. Furthermore, in the presence of 100 nM AG879, a tyrosine kinase phosphorylation inhibitor specific to TRKA, but not TRKB or TRKC, A1EVs still promoted neuron survival. At much higher levels of inhibitor (5 µM), reduced apoptosis levels were observed in A1-EV-treated neurons indicating that A1-EVs can rescue neuron survival independent of the TRKA receptor.

Taken together, these data indicate that A1EVs drive neurite growth and neuron survival through activation of the NGF/ERK5 signaling pathway but can occur independent of TRKA receptor activation. Further studies are required to identify if other receptors are involved, and which A1-EV cargo are fully responsible for the enhanced neurite growth and neuron survival observed.

While the present findings reveal salutary effects of A1EVs on neurite outgrowth and survival of mammalian SCG neurons, extensive new experiments are required to establish whether newt EVs are worthy of development as therapeutic candidates for neurodegenerative diseases and/or ischemic stroke. We have not shown that control EVs derived from intact newt skeletal muscle are effective, as would be needed to rule out the functionality of EV generation inhibitors. On the positive side, we have shown intriguing benefits on neuronal growth and survival in vitro. Moreover, EVs are capable of crossing the blood–brain barrier^53,54^, making systemic delivery potentially feasible. On the other hand, it would not be surprising if newt EVs turn out to be immunogenic in mammals, particularly with repeated exposures. Regardless, the fact that EVs from amphibians are bioactive in mammalian neurons is noteworthy, speaking to the fundamental conservation of recruitable regenerative pathways despite millennia of divergent evolution.

## Methods

### Newt cell culture

A1 cells, derived from normal hind limb muscle of *Notophthalmus viridescens*, were kindly provided by Dr Craig M. Crews (Yale University). The identity of the cells was confirmed based on morphology and the expression of blastema-associated keratin, as identified using the 22/17 mouse monoclonal antibody^55,56^. A1 cells were cultured on gelatin-coated (0.75% w/v) flasks in Newt Media (dilute minimum essential media (70% MEM + 30% deionized water) containing 10% heat-inactivated fetal bovine serum (FBS; Gibco), 100 U/mL antibiotics (Penicillin/Streptomycin, Gibco) and 2 mM l-glutamine (Gibco)^56^. Although insulin is commonly added to newt cell cultures to increase proliferation, no insulin was used in this study in order to prevent insulin carry-over into the neuron cultures. The cells were cultured at 25 °C in 2.5% CO_2_.

### EV isolation and EV inhibitor treatment methods

To generate EVs, A1 cells were grown to ~ 80% confluency in newt cell media. Next, the media was removed, and the newt cells were washed one time in Newt PBS (80% 1 × PBS + 20% deionized water) and then replaced with diluted (70%) MEM only, for 48 h. After 2 days, EVs were isolated by collecting the media from the A1 cells and centrifuging at 4000×*g* for 20 min to remove cell debris. Then the media was centrifuged again at 15,000×*g* for 30 min to remove larger particulates from the solution. The isolated media was filtered through a 450 µm mesh filter (Stericup, Millipore) to remove larger vesicles and large protein aggregates. Finally, EVs were isolated from the remaining media by passing the culture media through a 100 kDa filter (Amicon, Ultra 70), followed by 2 × 50 mL of sterile PBS to wash away culture media containing secreted growth factors, such as NGF, or any residual EV inhibitors that may have remained while still retaining the EVs. Centrifugation of the inverted filter removed the concentrated EVs in solution (approximately tenfold compared to culture media) from the filter. The concentrated EVs were stored short-term at 4 °C.

### Suppression of EV synthesis

To suppress EV generation in the newt cells, A1 cultures, grown in complete newt media, were exposed to different EV generation inhibitors (GW4869, Cambinol or Ketotifen fumarate in dimethyl sulfoxide (DMSO)) for 24 h at the time of cell plating, and prior to serum starvation. Inhibitor exposure at the time of cell plating allowed for the use of much lower inhibitor concentrations on these slow growing cells. After 24 h, the media was removed from the A1 cells, and then the cells were washed once with newt PBS and then cultured in dilute MEM (70%) for 48 h. Any EVs present in the culture media were collected as described above. Quantification of EV particle size distribution and amount was conducted using the Nanosight NS300 (Malvern).

### PC-12 cell culture

PC-12 cells (ATCC CRL-1722) were cultured in RPMI 1640 (Gibco) plus 10% heat-inactivated horse serum and 5% fetal bovine serum (Sigma) + 10 ng/mL Nerve Growth Factor (NGF, Sigma). The cells were plated on fibronectin-coated, 6-well culture dishes for 12 h and then treated with 1 × 10^8^ A1EVs/mL or the equivalent volume of A1 culture media lacking EVs (20 µM GW4869; as described above) or media-only control for 24 h. After 24 h post-treatment, the PC-12 cells were imaged via phase-contrast microscopy (Axiovert 200M, Zeiss). Cells with neurite-like extensions longer than one soma length were counted as positive and analysist were blinded to treatment group information.

### Neuron isolation and culture

To obtain sympathetic neurons, superior cervical ganglia (SCG) were isolated from behind the carotid artery from 2-day old Sprague–Dawley rat pups^57^. To collect rat sensory neurons, dorsal root ganglia (DRG) were isolated from the excised cervical vertebrae as described in mouse^58^. After harvesting, the neural tissue and meniscus surrounding the DRGs were removed. Both ganglia types were digested for 30 min at 37 °C using collagenase 2 (304U) in PBS, followed by 10 min of digestion with 0.1% trypsin. The digestion media was neutralized using complete neural media (Neurobasal media (Gibco), 2% B27 without insulin (Gibco), 1% Pen/Strep, 1% l-Glutamine) + 20% heat-inactivated FBS and centrifuged. After removing the media, the pellet was resuspended in complete neural media (no FBS) plus the addition of nerve growth factor (NGF); 10 ng/mL for the SCG neurons and 50 ng/mL for the DRG neurons. Neurons isolated from the ganglia were plated on poly-d-lysine (200 μg/mL) and laminin (10 μg/mL)-coated, 25 mm glass coverslips in 300 μL of media and placed inside 6-well culture plates. After an hour of incubation at 37 °C, 1 mL of complete neural media plus NGF was placed in each well and the plates were further incubated at 37 °C. The number of neurons plated is specific to each experiment.

### Neurite growth and Sholl analysis

To measure sympathetic neurite growth and branching, SCG neurons were seeded onto PDL/laminin-coated, 25 mm coverslips at 35,000 cells per coverslip in 300 μL of media for 1 h. Then 1 mL of complete neural media containing A1EVs or media alone was added to the cultures inside a 6-well dish. Neurons were cultured for 12 h and then rinsed and fixed with 4% paraformaldehyde in PBS plus 0.1% Tween 20 (PBST). Neurons were stained with mouse anti-acetylated-tubulin primary antibody (1:100, Abcam) followed by anti-mouse FITC secondary (1:400, Alexafluor 488, ThermoFisher). Confocal microscopy (Leica SP5) was used to image the neurons and the analysis was conducted using the *Simple Neurite Tracer* on ImageJ (NIH). To measure neurite length and number of neurite intersections, in actively growing neurons, only neurons not in contact with neighboring cell or neurites were measured. Greater than 50 neurons were counted per treatment.

### NGF deprivation apoptosis study and TUNEL staining

SCG neurons were seeded at 50,000 cells per coverslip and grown for 48 h in complete neural media + 10 ng/mL NGF. After 48 h, the established neurons were washed once in warm PBS and then complete neural media with NGF (positive control) or without NGF was added to the neuron cultures. Additionally, A1EVs (1 × 10^8^) were added to designated neuron cultures at the time of media change. After 12 h, the neurons were fixed in 4% PFA for 10 min and apoptotic cells were identified using terminal deoxynucleotidyl transferase biotin-dUTP nick end labeling (TUNEL). To identify neurons, mouse anti-acetylated-tubulin primary antibody (1:100, Abcam) followed by anti-mouse far red secondary antibody (1:400, Alexafluor 647, ThermoFisher) were used. DAPI was used to stain nuclei. Images were taken via Leica SP5 confocal microscope (Leica, SP5). Apoptotic nuclei were counted using ImageJ (NIH). For experiments involving suppression of the tropomyosin receptor kinase A (TRKA) receptor, the TRK A pathway competitive inhibitor, AG879 (100 nM), was added to the neuron cultures concurrently with the media change^29,30^.

### Blockage of transcription in SCG neurons

The A1EV-mediated transfer of mRNAs to the SCG neurons was evaluated by RT-qPCR performed on total RNA extracted from SCG neurons treated with 1 × 10^8^ A1EVs/mL or media-only control, in the presence of actinomycin D (ActD; Sigma). SCG neurons were pretreated with Actinomycin D (10 µg/mL, Sigma)^59^ for 3 h to inhibit RNA transcription. After thorough washing in sterile PBS, the SCG neurons were incubated with A1EVs or media control for 6 h, and then harvested and the RNA isolated (RNEasy Mini kit, Qiagen). Following reverse transcription of the RNA, the cDNA was subjected to RT-qPCR to quantify RUNX3 mRNA.

### Cellular respirometry

The XFe 96 extracellular flux analyzer (Seahorse Biosciences) was used to measure cellular respiration in neurons, as described^60^. Approximately 10,000 neonatal rat primary sympathetic neurons, isolated from the superior cervical ganglia, were seeded into each well of the XFe 96 cell culture microplates. Plates were incubated for 24 h at 37 °C in complete neural media. After 24 h, the media was removed and cells were washed in warm PBS and then neural media without FBS was added to the cells containing 1 × 10^8^ A1EVs/mL, or equivalent volumes of minimal media only, or equivalent volumes of A1 cell culture media whose EV generation had been suppressed with increasing concentrations of GW4869, as described above. Following 12 h of incubation at 37 °C, the cell media was replaced with XF Assay Medium (Seahorse Bioscience), supplemented with 10 mM glucose, 2 µM l-glutamine, and 1 mM sodium pyruvate (pH 7.4), and incubated at 37 °C in a non-CO_2_ incubator. The sensor cartridge was loaded with 1 µM Oligomycin, 1 µM FCCP, and a mixture of 1 µM antimycin and 1 µM rotenone. The XFe 96 conducted the standard mitochondrial stress test and we report assessments of mitochondrial respiration, basal respiration rates, spare respiratory capacity, proton leak, and ATP production.

### Western blot analysis

Cellular lysates of SCG neurons, treated with media only or 1 × 10^8^ A1EVs/mL, were generated using radioimmunoprecipitation assay buffer (RIPA) lysis buffer (Thermo) plus the HALT protease inhibitor cocktail (Thermo) and prepared for sodium dodecyl sulfate polyacrylamide gel electrophoresis (SDS PAGE) separation. Protein quantification of the lysates was performed using a BCA protein assay kit (Thermo Scientific). A total amount of 30 μg of proteins per well was separated on 4–12% SDS–polyacrylamide gels in quadruplicate and transferred onto nitrocellulose blotting membranes (Bio-Rad). Blocking of the membranes was performed using 5% non-fat milk in PBS containing 0.5% Tween 20. Neuron lysate blots were probed using the following primary antibodies: RUNX3 (1:1000 Fisher Scientific), TOM70 (1:100, Sigma), TrkA (1:1000, Novus Biologicals), ERK5 (1:1000, Novus Biologicals), p-ERK5 (1:1000, Fisher Scientific), CREB (1:1000, Novus Biologica), p-CREB (1:1000, Novus Biologica), β-actin (1:5000, Thermo Fishier Scientific). Following washing with TBS-Tween buffer and incubation with appropriate HRP-labelled secondary antibodies, protein detections were performed using ECL kit (Thermo Scientific). Blots were imaged using the Gel Doc XR+ system (BioRad) and analyzed using ImageJ (NIH).

### A1EV fluorescence tracking

A1 cells at ~ 80% confluency were transfected with a plasmid that generates a fusion protein of Green Fluorescent protein (GFP) and the tetraspanin protein, CD63, under the control of the CMV promoter (pCT-CD63-GFP, System Biosciences # CYTO120-PA-1). 2.5 μg of plasmid in 200 μL of serum-free, dilute MEM (70%) was mixed with 6 μL of transfection reagent (Pure-fection, System Biosciences) and added to 1.8 mL of complete newt media. Each well of the 6-well plate was incubated in 2 mL of the reagent media for 24 h and then rinsed in newt PBS and complete newt media was added. Approximately half of the A1 cells expressed GFP at 48 h post treatment. After 24 h in complete newt media, the A1 cells were serum-starved as described above. GFP-tagged A1EVs were generated and isolated as described above. For the A1EV tracking study, 5 × 10^8^/mL GFP-labelled A1EVs, were added to SCG neurons in culture for the time described. Treated neurons were fixed in 4% PFA and stained against mouse anti-rat alpha acetylated tubulin (1:300, Sigma), rabbit anti-rat RAB7 (1:100, Abcam) and chicken anti-GFP (1:200, Abcam) primary antibodies and anti-mouse Alexa fluor 647, anti-rabbit Alexa fluor 555, and anti-chicken Alexa fluor 488 secondary antibodies respectively (1:400, Thermo). Images were taken using the Leica TCS SP5 confocal microscope.

### RNA preparation and next generation sequencing and analysis

RNA was extracted from neonatal rat neuron lysate (Qiazol) using the RNeasy Mini kit (Qiagen) per manufacturer’s instructions. The total RNA yields were quantitated by NanoDrop A260 for overall recoveries and RNA sequencing was performed (Illumna). Ingenuity Pathway Analysis (IPA, Qiagen) was used to identify the canonical pathways activated in A1-EV-treated neurons relative to media only-treated neurons. Genes with an absolute log2 fold change (|log2 fold change|) higher than 2.5 and p-value < 0.05 compared to the control samples were defined as differentially expressed and were analyzed by the IPA software (version 2021; Ingenuity Systems; QIAGEN). DEGs from RNA sequencing outcomes were uploaded to IPA for core analysis with the global molecular network in the ingenuity pathway knowledge base (IPKB)^61–63^. Canonical signaling pathways enriched by the DEGs were identified and rated according to p-values.

### Gene ontology analysis

Gene Ontology enRIchment anaLysis and visuaLizAtion tool tool (Gorilla67)^64,65^ was used to identify enriched GO terms related to biological processes from gene lists ranked by increasing or decreasing Pearson correlation with NE scores in cell line data sets. All mRNA identified in A1-derived Evs^18^ was used to populate the ranked lists for this study. The biological processes and cellular components and their respective p-values, FDR q-values, and enrichment scores can be found within the supplementary tables.

### Statistical analysis

Data are presented as mean ± SEM. One-way ANOVA or Student’s unpaired *t*-test was used for comparisons between groups. A value of p < 0.05 was considered significant. p-value thresholds are described within the figure legends.

## Supplementary Information

Supplementary Information.

Supplementary Table 1.

Supplementary Table 2.

## Data Availability

The datasets generated during and/or analyzed during the current study are available from the corresponding author on reasonable request.
