# Supplementary material for: Newt A1 cell-derived extracellular vesicles promote mammalian nerve growth
Source: Sci Rep. 2023 Jul 22;13:11829. doi: 10.1038/s41598-023-38671-z (PMC10363125; doi:10.1038/s41598-023-38671-z)
Supplement: Supplementary file 1 — Supplementary Information. [file 41598_2023_38671_MOESM1_ESM.docx]

**Supplemental Figure 1**


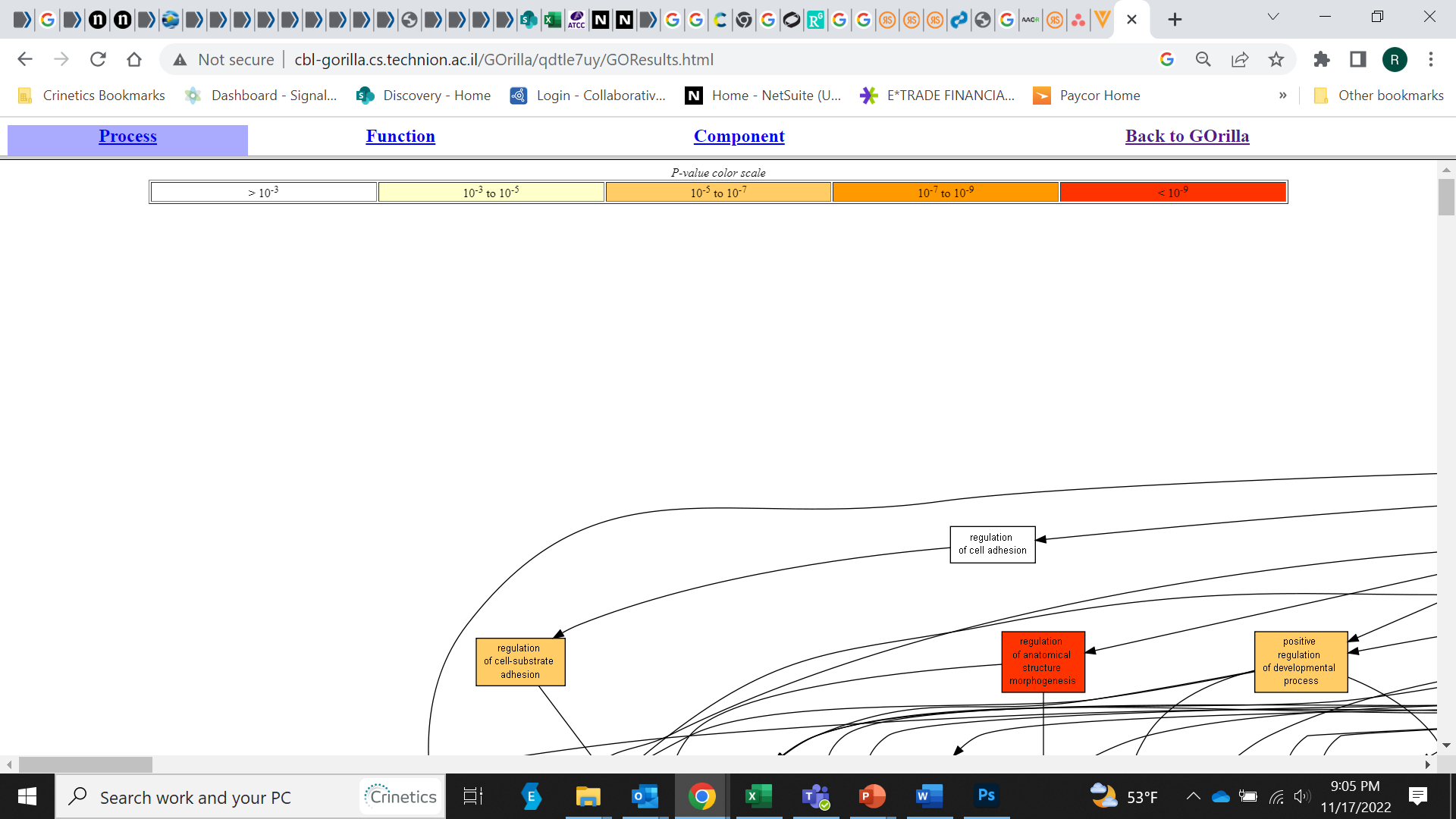


**Supplemental Figure 1: *In Silico* analysis of newt A1EV cargo identifies genes involved in neural growth and development.** The biological processes of Gene ontology analysis of all abundance-ranked mRNAs identified within A1-EVs are shown. The red box indicates the subsection of the GOA map shown in Figure 1c.

**Supplemental Table 1**

Gene Ontology Analysis Table containing the enrichment p-values, total number of genes in the analysis, and the number of genes associated with each specific GO term as grouped by Biological Processes.

**Supplemental Figure 2**


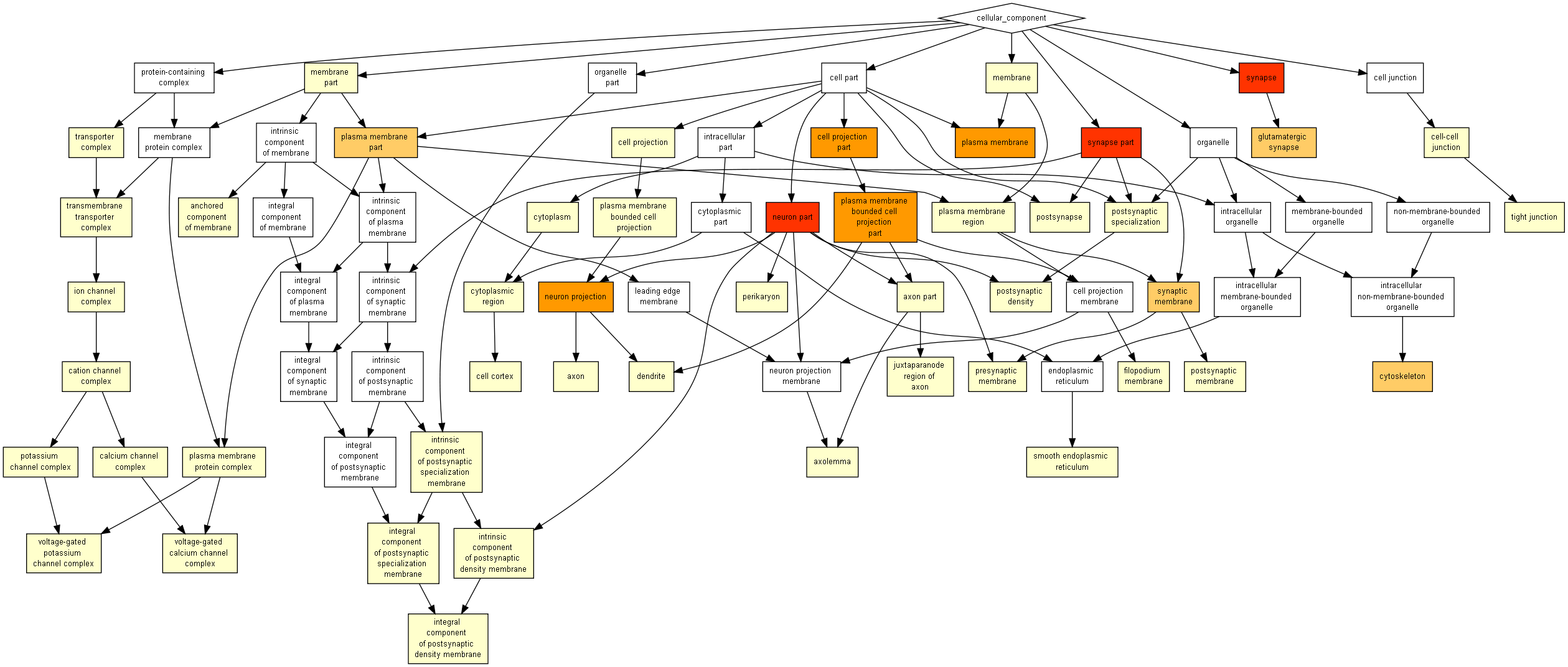


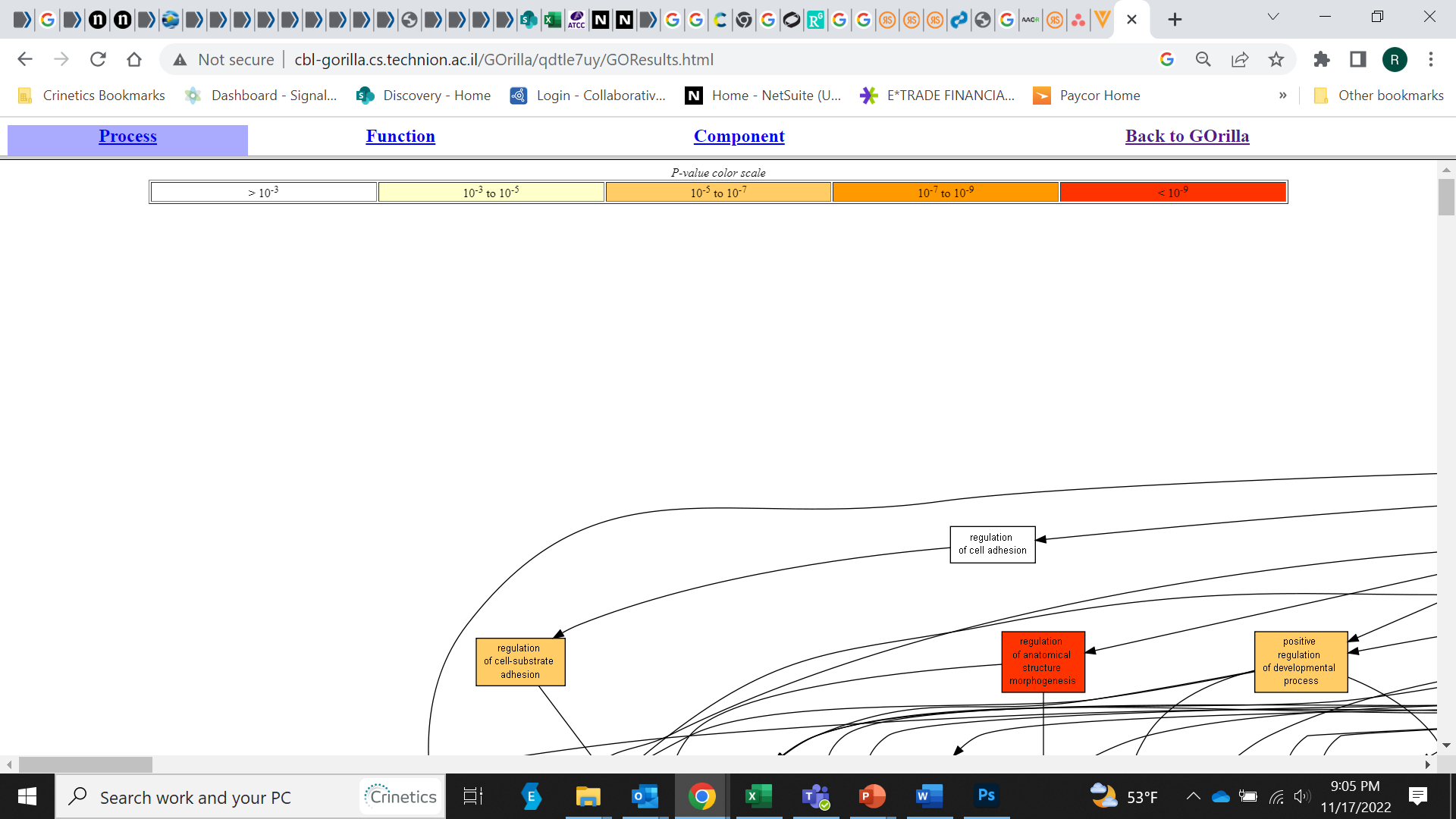


**Supplemental Figure 2: *In Silico* analysis of newt A1EV cargo identifies genes involved in neural growth and development.** Cellular components identified by Gene ontology analysis of all abundance-ranked mRNAs identified within A1-EVs are shown.

**Supplemental Table 2**

Gene Ontology Analysis Table containing the enrichment p-values, total number of genes in the analysis, and the number of genes associated with each specific GO term as grouped by Cellular Components.

**Supplemental Figure 3**


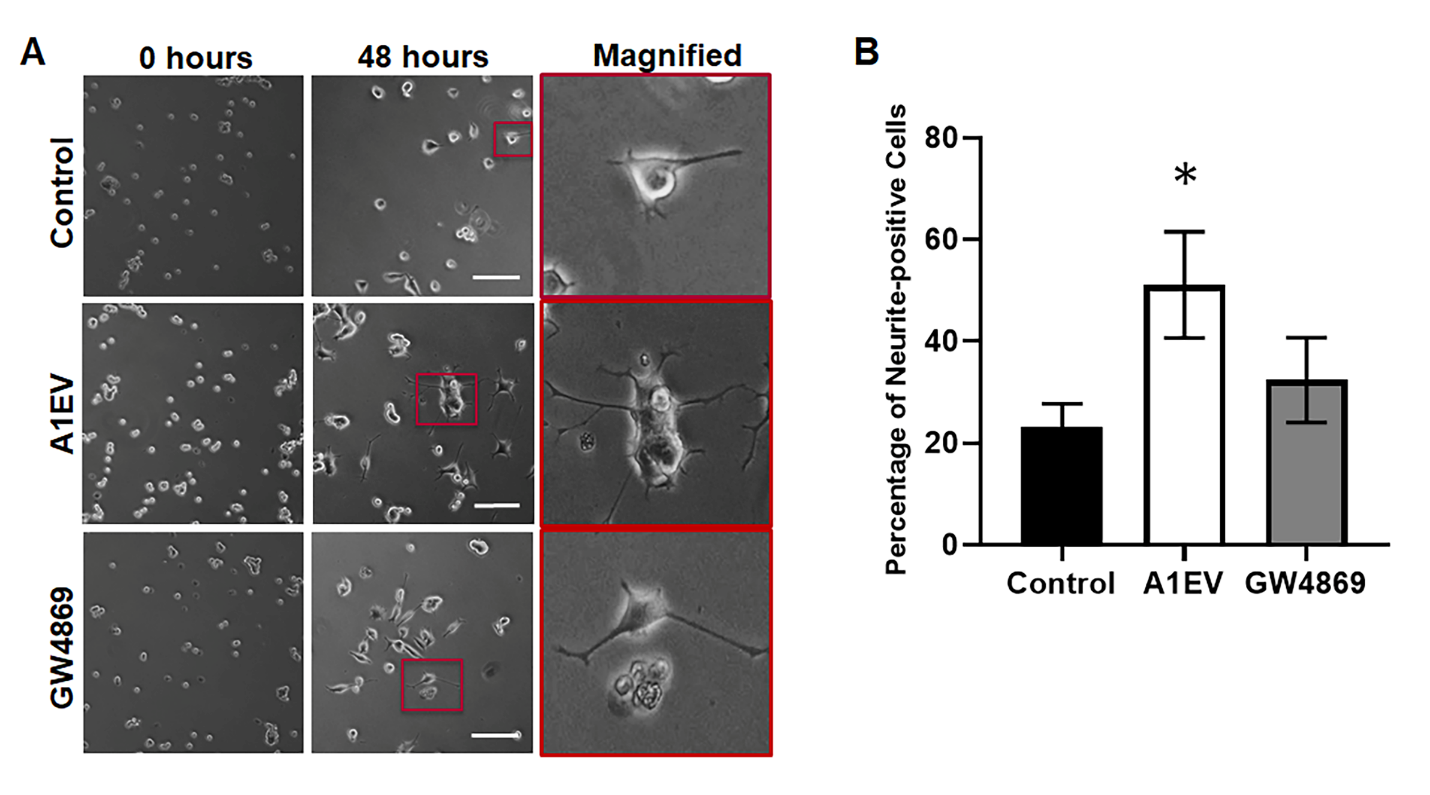


**Supplemental Figure 3: A1EVs drive neurite-like extensions in cell lines that model nerve growth.** A) Phase-contrast microscopy images of PC-12 cells shown immediately prior to treatment and 48 hours post-treatment, with cell culture media only, 1x10^8^ A1/EVs, or an equivalent volume of A1 culture media where EV generation has been suppressed (A1-GW4869). Scale bar = 25µm. 10x magnification of the areas within the red squares are shown in the third column. B) Graphical representation of neurite-positive cells in culture at 48 hours post treatment. * indicates statistical significance, p < 0.05.

**Supplemental Figure 4:**


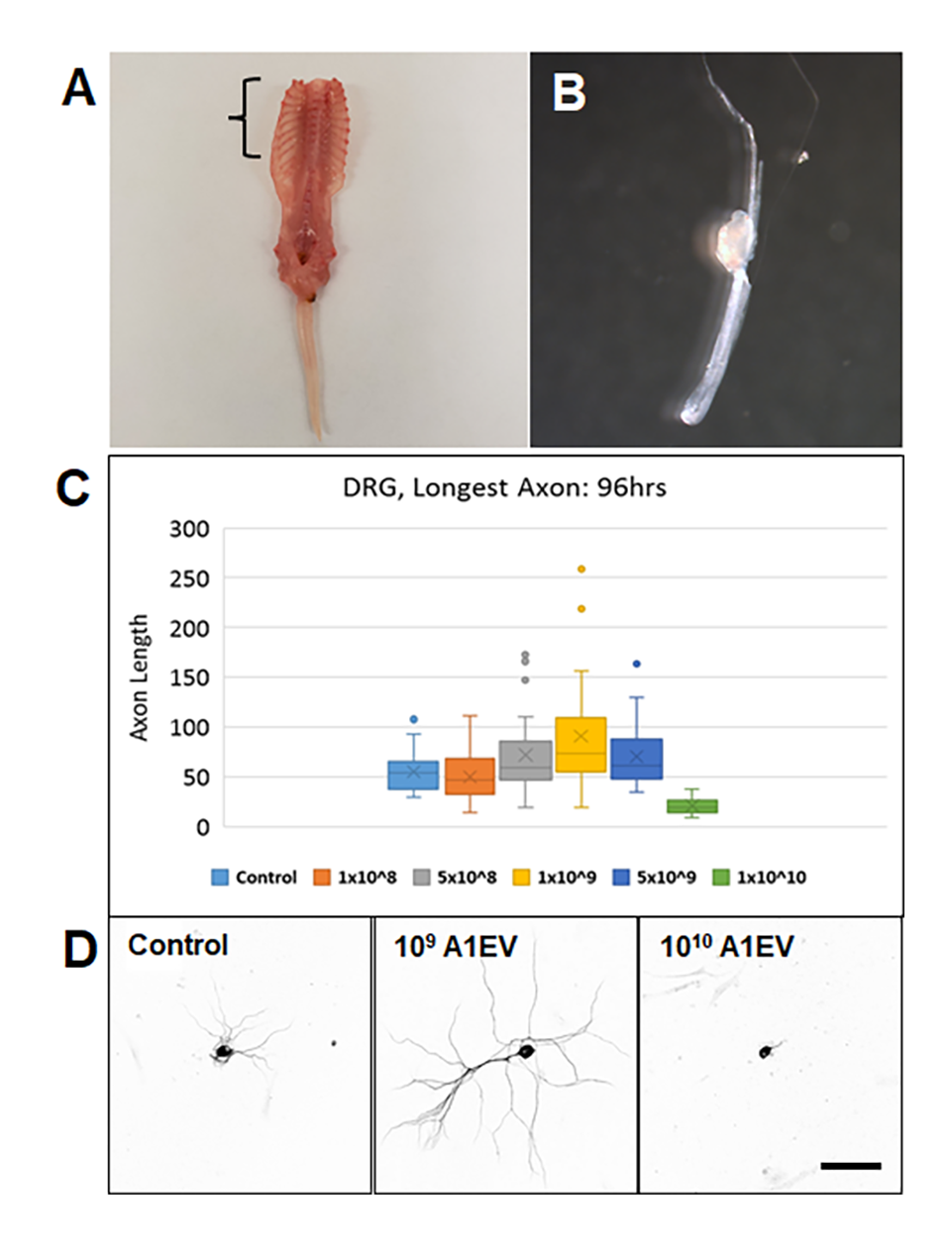


**Supplemental Figure 4: A1EV exposure drives neurite growth in Dorsal Root Ganglion-derived sensory neurons in culture.** A) Spinal location of the source of DRGs isolated from 3 day old rat pups. B) Isolated DRG with nerve root and meniscus still attached. C) Graphical representation of DRG neuron length following 96-hour culture in increasing concentrations of A1EVs. D) Example images of DRG neuron silhouettes show neurite length treated with different A1EV concentrations. Graphs and images indicate concentrations as EV number per milliliter.
