# Supplementary material for: Newt A1 cell-derived extracellular vesicles promote mammalian nerve growth
Source: Sci Rep. 2023 Jul 22;13:11829. doi: 10.1038/s41598-023-38671-z (PMC10363125; doi:10.1038/s41598-023-38671-z)
Supplement: Supplementary file 2 — Supplementary Table 1. [file 41598_2023_38671_MOESM2_ESM.pdf]

GO term	Description	P-value	FDR q-value	Enrichment (N, B, n, b)
GO:0007155	cell adhesion	2.16E-10	2.45E-06	2.93 (5713,371,242,46)
GO:0022610	biological adhesion	2.33E-10	1.32E-06	2.92 (5713,372,242,46)
GO:0010975	regulation of neuron projection development	1.64E-09	6.20E-06	2.26 (5713,245,650,63)
GO:0120035	regulation of plasma membrane bounded cell projection organization	3.32E-09	9.42E-06	2.27 (5713,320,487,62)
GO:0031344	regulation of cell projection organization	3.32E-09	7.54E-06	2.27 (5713,320,487,62)
GO:0034762	regulation of transmembrane transport	2.04E-08	3.87E-05	2.36 (5713,211,584,51)
GO:0034765	regulation of ion transmembrane transport	2.91E-08	4.72E-05	2.05 (5713,185,948,63)
GO:0060284	regulation of cell development	3.32E-08	4.72E-05	1.68 (5713,384,981,111)
GO:0098609	cell-cell adhesion	3.91E-08	4.93E-05	5.03 (5713,198,109,19)
GO:0051960	regulation of nervous system development	4.23E-08	4.80E-05	1.68 (5713,381,981,110)
GO:0050767	regulation of neurogenesis	4.53E-08	4.67E-05	1.72 (5713,341,981,101)
GO:0045664	regulation of neuron differentiation	4.82E-08	4.56E-05	2.04 (5713,289,650,67)
GO:0098742	cell-cell adhesion via plasma-membrane adhesion molecules	4.88E-08	4.26E-05	7.62 (5713,99,106,14)
GO:0099177	regulation of trans-synaptic signaling	6.25E-08	5.07E-05	2.01 (5713,195,933,64)
GO:0050804	modulation of chemical synaptic transmission	6.25E-08	4.73E-05	2.01 (5713,195,933,64)
GO:0008038	neuron recognition	1.07E-07	7.58E-05	4.92 (5713,23,757,15)
GO:1904062	regulation of cation transmembrane transport	1.30E-07	8.67E-05	2.55 (5713,139,662,41)
GO:0048731	system development	1.36E-07	8.61E-05	2.02 (5713,266,680,64)
GO:0043269	regulation of ion transport	1.74E-07	1.04E-04	2.17 (5713,243,584,54)
GO:0051962	positive regulation of nervous system development	2.52E-07	1.43E-04	2.14 (5713,219,659,54)
GO:0032412	regulation of ion transmembrane transporter activity	6.30E-07	3.41E-04	2.67 (5713,110,662,34)

GO:0023052	signaling	6.83E-07	3.52E-04	1.98 (5713,196,852,58)
GO:0022898	regulation of transmembrane transporter activity	7.98E-07	3.94E-04	2.64 (5713,111,662,34)
GO:0050807	regulation of synapse organization	1.05E-06	4.96E-04	2.91 (5713,101,563,29)
GO:0032409	regulation of transporter activity	1.59E-06	7.24E-04	2.53 (5713,117,675,35)
GO:0050773	regulation of dendrite development	1.63E-06	7.13E-04	2.39 (5713,88,978,36)
GO:0032879	regulation of localization	1.70E-06	7.17E-04	1.59 (5713,956,418,111)
GO:0044057	regulation of system process	2.46E-06	9.98E-04	2.12 (5713,188,675,47)
GO:0050808	synapse organization	2.47E-06	9.66E-04	4.46 (5713,90,242,17)
GO:0022604	regulation of cell morphogenesis	2.63E-06	9.96E-04	1.77 (5713,234,978,71)
GO:0045666	positive regulation of neuron differentiation	2.99E-06	1.09E-03	2.20 (5713,168,648,42)
GO:0010976	positive regulation of neuron projection development	3.33E-06	1.18E-03	2.38 (5713,141,648,38)
GO:0050769	positive regulation of neurogenesis	4.10E-06	1.41E-03	2.08 (5713,199,648,47)
GO:0031346	positive regulation of cell projection organization	4.41E-06	1.47E-03	3.12 (5713,184,239,24)
GO:0010720	positive regulation of cell development	5.65E-06	1.83E-03	2.00 (5713,225,648,51)
GO:0051270	regulation of cellular component movement	8.07E-06	2.54E-03	1.58 (5713,363,977,98)
GO:0010769	regulation of cell morphogenesis involved in differentiation	8.25E-06	2.53E-03	2.00 (5713,148,946,49)
GO:0042391	regulation of membrane potential	8.34E-06	2.49E-03	1.87 (5713,177,948,55)
GO:0099536	synaptic signaling	9.44E-06	2.75E-03	2.18 (5713,123,852,40)
GO:2001257	regulation of cation channel activity	1.00E-05	2.85E-03	2.58 (5713,75,827,28)
GO:0002028	regulation of sodium ion transport	1.12E-05	3.11E-03	3.01 (5713,40,948,20)
GO:0032502	developmental process	1.27E-05	3.43E-03	1.37 (5713,1686,416,168)
GO:0030334	regulation of cell migration	1.34E-05	3.55E-03	1.62 (5713,307,977,85)

GO:0007416	synapse assembly	1.37E-05	3.54E-03	3.51 (5713,31,839,16)
GO:0048638	regulation of developmental growth	1.43E-05	3.60E-03	2.09 (5713,124,928,42)
GO:0051963	regulation of synapse assembly	1.53E-05	3.77E-03	3.78 (5713,43,563,16)
GO:0099537	trans-synaptic signaling	1.68E-05	4.05E-03	2.16 (5713,121,852,39)
GO:0009653	anatomical structure morphogenesis	1.68E-05	3.97E-03	1.68 (5713,497,540,79)
GO:0007267	cell-cell signaling	2.51E-05	5.82E-03	1.96 (5713,164,852,48)
GO:0040008	regulation of growth	2.92E-05	6.62E-03	1.79 (5713,222,833,58)
GO:0051128	regulation of cellular component organization	3.44E-05	7.66E-03	1.44 (5713,1011,513,131)
GO:0001952	regulation of cell-matrix adhesion	3.54E-05	7.74E-03	3.20 (5713,63,538,19)
GO:0051965	positive regulation of synapse assembly	3.75E-05	8.04E-03	5.53 (5713,25,413,10)
GO:2000145	regulation of cell motility	3.87E-05	8.14E-03	1.57 (5713,328,977,88)
GO:2000146	negative regulation of cell motility	4.14E-05	8.54E-03	2.15 (5713,96,968,35)
GO:0099174	regulation of presynapse organization	4.19E-05	8.50E-03	29.26 (5713,11,71,4)
GO:1905606	regulation of presynapse assembly	4.19E-05	8.35E-03	29.26 (5713,11,71,4)
GO:2000463	positive regulation of excitatory postsynaptic potential	5.34E-05	1.04E-02	6.34 (5713,14,515,8)
GO:0030010	establishment of cell polarity	5.43E-05	1.05E-02	2.70 (5713,47,944,21)
GO:0021932	hindbrain radial glia guided cell migration	5.47E-05	1.04E-02	43.28 (5713,4,99,3)
GO:0021942	radial glia guided migration of Purkinje cell	5.47E-05	1.02E-02	43.28 (5713,4,99,3)
GO:0007156	homophilic cell adhesion via plasma membrane adhesion molecules	5.84E-05	1.07E-02	5.35 (5713,66,178,11)
GO:0007399	nervous system development	6.11E-05	1.10E-02	2.19 (5713,137,665,35)
GO:0010959	regulation of metal ion transport	6.89E-05	1.22E-02	2.21 (5713,131,670,34)
GO:0040012	regulation of locomotion	6.91E-05	1.21E-02	1.53 (5713,347,977,91)

GO:2000649	regulation of sodium ion transmembrane transporter activity	7.09E-05	1.22E-02	4.08 (5713,26,646,12)
GO:0007163	establishment or maintenance of cell polarity	8.02E-05	1.36E-02	2.24 (5713,81,944,30)
GO:0048639	positive regulation of developmental growth	8.05E-05	1.34E-02	2.63 (5713,69,724,23)
GO:0051049	regulation of transport	9.39E-05	1.54E-02	1.64 (5713,607,425,74)
GO:1902305	regulation of sodium ion transmembrane transport	1.08E-04	1.75E-02	3.02 (5713,32,946,16)
GO:0007154	cell communication	1.10E-04	1.76E-02	1.66 (5713,262,852,65)
GO:0060998	regulation of dendritic spine development	1.11E-04	1.75E-02	2.66 (5713,44,978,20)
GO:0051271	negative regulation of cellular component movement	1.20E-04	1.87E-02	2.02 (5713,108,968,37)
GO:0048814	regulation of dendrite morphogenesis	1.29E-04	1.97E-02	2.59 (5713,49,946,21)
GO:0098916	anterograde trans-synaptic signaling	1.44E-04	2.19E-02	2.08 (5713,113,852,35)
GO:0007268	chemical synaptic transmission	1.44E-04	2.16E-02	2.08 (5713,113,852,35)
GO:0046716	muscle cell cellular homeostasis	1.59E-04	2.34E-02	39.67 (5713,6,72,3)
GO:0061502	early endosome to recycling endosome transport	1.75E-04	2.55E-02	5,713.00 (5713,1,1,1)
GO:0007157	heterophilic cell-cell adhesion via plasma membrane cell adhesion molecules	1.76E-04	2.53E-02	5.17 (5713,22,452,9)
GO:0030336	negative regulation of cell migration	1.77E-04	2.51E-02	2.10 (5713,90,968,32)
GO:0040013	negative regulation of locomotion	1.84E-04	2.59E-02	1.99 (5713,110,968,37)
GO:0021535	cell migration in hindbrain	1.85E-04	2.56E-02	8.67 (5713,6,549,5)
GO:1900006	positive regulation of dendrite development	1.89E-04	2.58E-02	4.47 (5713,42,335,11)
GO:0032989	cellular component morphogenesis	1.93E-04	2.61E-02	2.03 (5713,213,476,36)
GO:0051705	multi-organism behavior	2.15E-04	2.87E-02	3.44 (5713,21,950,12)
GO:0035556	intracellular signal transduction	2.19E-04	2.89E-02	1.41 (5713,662,752,123)
GO:1904063	negative regulation of cation transmembrane transport	2.20E-04	2.87E-02	4.57 (5713,26,481,10)

GO:0048812	neuron projection morphogenesis	2.26E-04	2.92E-02	2.58 (5713,107,476,23)
GO:1901019	regulation of calcium ion transmembrane transporter activity	2.35E-04	3.00E-02	3.20 (5713,31,806,14)
GO:0050768	negative regulation of neurogenesis	2.40E-04	3.03E-02	2.00 (5713,102,981,35)
GO:0031646	positive regulation of neurological system process	2.47E-04	3.08E-02	4.44 (5713,25,515,10)
GO:0002090	regulation of receptor internalization	2.59E-04	3.20E-02	5.40 (5713,17,498,8)
GO:0031644	regulation of neurological system process	2.66E-04	3.25E-02	2.85 (5713,58,622,18)
GO:0022603	regulation of anatomical structure morphogenesis	2.79E-04	3.37E-02	1.67 (5713,412,516,62)
GO:0006937	regulation of muscle contraction	3.35E-04	4.01E-02	12.95 (5713,45,49,5)
GO:0048167	regulation of synaptic plasticity	3.81E-04	4.51E-02	2.58 (5713,81,574,21)
GO:0051094	positive regulation of developmental process	4.14E-04	4.85E-02	1.93 (5713,473,244,39)
GO:0045595	regulation of cell differentiation	4.16E-04	4.82E-02	1.34 (5713,633,981,146)
GO:0034763	negative regulation of transmembrane transport	4.17E-04	4.79E-02	3.96 (5713,33,481,11)
GO:0034766	negative regulation of ion transmembrane transport	4.26E-04	4.83E-02	4.24 (5713,28,481,10)
GO:0120039	plasma membrane bounded cell projection morphogenesis	4.27E-04	4.80E-02	2.49 (5713,111,476,23)
GO:0065009	regulation of molecular function	4.48E-04	4.99E-02	1.26 (5713,1150,877,222)
GO:0051130	positive regulation of cellular component organization	4.52E-04	4.98E-02	1.59 (5713,504,491,69)
GO:0060999	positive regulation of dendritic spine development	4.80E-04	5.24E-02	4.01 (5713,23,619,10)
GO:0001558	regulation of cell growth	4.82E-04	5.21E-02	1.88 (5713,146,833,40)
GO:0048858	cell projection morphogenesis	4.85E-04	5.20E-02	2.46 (5713,112,476,23)
GO:0099545	trans-synaptic signaling by trans-synaptic complex	5.06E-04	5.37E-02	105.80 (5713,6,18,2)
GO:0086004	regulation of cardiac muscle cell contraction	5.10E-04	5.36E-02	6.88 (5713,12,415,6)
GO:1903115	regulation of actin filament-based movement	5.10E-04	5.31E-02	6.88 (5713,12,415,6)

GO:0051239	regulation of multicellular organismal process	5.35E-04	5.52E-02	1.25 (5713,1068,981,229)
GO:0090162	establishment of epithelial cell polarity	5.59E-04	5.72E-02	5.77 (5713,16,433,7)
GO:0010810	regulation of cell-substrate adhesion	5.63E-04	5.71E-02	2.36 (5713,102,570,24)
GO:0051961	negative regulation of nervous system development	5.64E-04	5.67E-02	1.89 (5713,114,981,37)
GO:0051893	regulation of focal adhesion assembly	5.79E-04	5.77E-02	3.54 (5713,36,538,12)
GO:0090109	regulation of cell-substrate junction assembly	5.79E-04	5.72E-02	3.54 (5713,36,538,12)
GO:0055117	regulation of cardiac muscle contraction	5.82E-04	5.70E-02	17.27 (5713,27,49,4)
GO:0010766	negative regulation of sodium ion transport	5.85E-04	5.68E-02	6.48 (5713,11,481,6)
GO:2000026	regulation of multicellular organismal development	6.30E-04	6.06E-02	1.46 (5713,730,516,96)
GO:0006942	regulation of striated muscle contraction	6.92E-04	6.60E-02	16.66 (5713,28,49,4)
GO:0099642	retrograde axonal protein transport	7.00E-04	6.62E-02	1,428.25 (5713,1,4,1)
GO:0098815	modulation of excitatory postsynaptic potential	7.10E-04	6.66E-02	3.85 (5713,24,619,10)
GO:0010977	negative regulation of neuron projection development	7.12E-04	6.62E-02	2.57 (5713,65,650,19)
GO:0045927	positive regulation of growth	7.12E-04	6.58E-02	2.11 (5713,94,807,28)
GO:0030900	forebrain development	7.50E-04	6.87E-02	4.80 (5713,20,476,8)
GO:0050793	regulation of developmental process	7.55E-04	6.86E-02	1.27 (5713,910,981,198)
GO:0032990	cell part morphogenesis	7.69E-04	6.93E-02	2.34 (5713,123,476,24)
GO:0045597	positive regulation of cell differentiation	8.07E-04	7.22E-02	1.61 (5713,344,631,61)
GO:1902306	negative regulation of sodium ion transmembrane transport	8.09E-04	7.18E-02	7.42 (5713,8,481,5)
GO:0010842	retina layer formation	8.12E-04	7.14E-02	13.60 (5713,12,140,4)
GO:0097105	presynaptic membrane assembly	8.23E-04	7.19E-02	90.68 (5713,7,18,2)
GO:0097090	presynaptic membrane organization	8.23E-04	7.13E-02	90.68 (5713,7,18,2)

GO:0045920	negative regulation of exocytosis	8.23E-04	7.08E-02	90.68 (5713,7,18,2)
GO:0097485	neuron projection guidance	8.29E-04	7.07E-02	2.06 (5713,121,686,30)
GO:0007411	axon guidance	8.29E-04	7.02E-02	2.06 (5713,121,686,30)
GO:0030307	positive regulation of cell growth	8.56E-04	7.20E-02	2.36 (5713,63,807,21)
GO:0050775	positive regulation of dendrite morphogenesis	8.65E-04	7.22E-02	29.10 (5713,19,31,3)
GO:0045163	clustering of voltage-gated potassium channels	8.75E-04	7.25E-02	1,142.60 (5713,1,5,1)
GO:0021761	limbic system development	8.75E-04	7.20E-02	1,142.60 (5713,1,5,1)
GO:0097120	receptor localization to synapse	9.43E-04	7.70E-02	3.32 (5713,23,822,11)
GO:0070050	neuron cellular homeostasis	9.72E-04	7.88E-02	11.76 (5713,9,216,4)
GO:0031345	negative regulation of cell projection organization	9.73E-04	7.83E-02	2.40 (5713,77,650,21)
GO:0021707	cerebellar granule cell differentiation	9.99E-04	7.99E-02	38.86 (5713,2,147,2)
