# Supplementary material for: Newt A1 cell-derived extracellular vesicles promote mammalian nerve growth
Source: Sci Rep. 2023 Jul 22;13:11829. doi: 10.1038/s41598-023-38671-z (PMC10363125; doi:10.1038/s41598-023-38671-z)
Supplement: Supplementary file 3 — Supplementary Table 2. [file 41598_2023_38671_MOESM3_ESM.pdf]

GO term	Description	P-value	FDR q-value	Enrichment (N, B, n, b)
GO:0045202	synapse	4.52E-13	7.03E-10	2.00 (5713,361,871,110)
GO:0097458	neuron part	2.37E-11	1.84E-08	1.72 (5713,775,636,148)
GO:0044456	synapse part	2.17E-10	1.12E-07	2.05 (5713,426,570,87)
GO:0005886	plasma membrane	2.24E-09	8.69E-07	1.35 (5713,1491,874,307)
GO:0120038	plasma membrane bounded cell projection part	5.74E-09	1.79E-06	1.52 (5713,696,949,176)
GO:0044463	cell projection part	5.74E-09	1.49E-06	1.52 (5713,696,949,176)
GO:0043005	neuron projection	8.86E-08	1.97E-05	1.62 (5713,448,963,122)
GO:0098978	glutamatergic synapse	4.05E-07	7.88E-05	2.10 (5713,158,931,54)
GO:0097060	synaptic membrane	6.36E-07	1.10E-04	2.99 (5713,138,416,30)
GO:0005856	cytoskeleton	7.47E-07	1.16E-04	1.91 (5713,415,489,68)
GO:0044459	plasma membrane part	2.01E-06	2.85E-04	1.77 (5713,998,242,75)
GO:0045211	postsynaptic membrane	1.05E-05	1.37E-03	4.19 (5713,75,291,16)
GO:0005938	cell cortex	1.07E-05	1.28E-03	2.30 (5713,89,949,34)
GO:0098590	plasma membrane region	1.48E-05	1.65E-03	1.71 (5713,461,543,75)
GO:0034704	calcium channel complex	1.61E-05	1.67E-03	3.13 (5713,41,846,19)
GO:0098797	plasma membrane protein complex	1.71E-05	1.67E-03	1.77 (5713,231,864,62)
GO:0042995	cell projection	2.04E-05	1.87E-03	1.37 (5713,771,966,178)
GO:0044224	juxtaparanode region of axon	2.08E-05	1.80E-03	457.04 (5713,5,5,2)
GO:0031225	anchored component of membrane	2.28E-05	1.87E-03	5.69 (5713,43,257,11)
GO:0120025	plasma membrane bounded cell projection	2.66E-05	2.07E-03	1.37 (5713,725,963,168)
GO:0098794	postsynapse	4.32E-05	3.20E-03	2.04 (5713,118,948,40)
GO:0008076	voltage-gated potassium channel complex	4.54E-05	3.21E-03	19.20 (5713,31,48,5)

GO:0033267	axon part	4.72E-05	3.19E-03	1.77 (5713,188,963,56)
GO:0005737	cytoplasm	6.03E-05	3.91E-03	1.23 (5713,1742,797,299)
GO:0030673	axolemma	9.09E-05	5.66E-03	285.65 (5713,8,5,2)
GO:0034705	potassium channel complex	9.89E-05	5.92E-03	16.53 (5713,36,48,5)
GO:0005891	voltage-gated calcium channel complex	1.14E-04	6.55E-03	3.51 (5713,25,846,13)
GO:0099568	cytoplasmic region	1.39E-04	7.72E-03	1.90 (5713,136,949,43)
GO:0043204	perikaryon	1.67E-04	8.96E-03	51.94 (5713,66,5,3)
GO:0070160	tight junction	1.86E-04	9.63E-03	6.86 (5713,56,119,8)
GO:0034702	ion channel complex	2.19E-04	1.10E-02	6.01 (5713,145,59,9)
GO:0034703	cation channel complex	2.30E-04	1.12E-02	6.92 (5713,112,59,8)
GO:0099146	intrinsic component of postsynaptic density membrane	2.51E-04	1.19E-02	3.35 (5713,27,822,13)
GO:0099061	integral component of postsynaptic density membrane	2.51E-04	1.15E-02	3.35 (5713,27,822,13)
GO:0005911	cell-cell junction	2.63E-04	1.17E-02	3.76 (5713,160,133,14)
GO:1902495	transmembrane transporter complex	2.67E-04	1.15E-02	5.89 (5713,148,59,9)
GO:0044425	membrane part	2.78E-04	1.17E-02	1.54 (5713,2026,117,64)
GO:0099572	postsynaptic specialization	3.47E-04	1.42E-02	2.21 (5713,119,631,29)
GO:1990351	transporter complex	3.78E-04	1.51E-02	5.62 (5713,155,59,9)
GO:0030424	axon	3.91E-04	1.52E-02	2.06 (5713,153,617,34)
GO:0099060	integral component of postsynaptic specialization membrane	5.31E-04	2.01E-02	2.90 (5713,36,822,15)
GO:0098948	intrinsic component of postsynaptic specialization membrane	5.31E-04	1.97E-02	2.90 (5713,36,822,15)
GO:0005790	smooth endoplasmic reticulum	6.32E-04	2.29E-02	5.90 (5713,9,646,6)

GO:0016020	membrane	6.47E-04	2.29E-02	1.20 (5713,2646,425,237)
GO:0031527	filopodium membrane	7.33E-04	2.53E-02	12.16 (5713,8,235,4)
GO:0030425	dendrite	7.41E-04	2.51E-02	1.62 (5713,199,977,55)
GO:0014069	postsynaptic density	7.60E-04	2.52E-02	2.15 (5713,118,631,28)
GO:0042734	presynaptic membrane	8.15E-04	2.64E-02	4.04 (5713,34,416,10)
